# Broadly neutralizing antibodies in adult males living with HIV undergoing analytical treatment interruption: secondary and exploratory outcomes of the phase II randomized controlled RIO trial

**DOI:** 10.1038/s41591-026-04644-8

**Published:** 2026-09-15

**Authors:** Marcilio J. Fumagalli, Anna Kaczynska, Marius Allombert, Ágata Lopes-Ribeiro, Mariângela de Oliveira Silva, Brianna J. Hernandez, Christy Lavine, Sebastian M. Espinosa, Helen Brown, Hanna Box, Emanuela Falaschetti, Louise-Rae Cherrill, Tamara Elliott, Ming Lee, Julie Fox, Sarah Pett, Amanda Clarke, Alison Uriel, Ole Sogaard, Rebecca Sutherland, Marta Boffito, Sabine Kinloch-Loes, Chloe Orkin, Simon Collins, Panagiota Zacharopoulou, Nicola Robison, Cintia Bittar, Thiago Oliveira, Anna Gazumyan, Mila Jankovic, Michael S. Seaman, John Frater, Marina Caskey, Sarah Fidler, Michel C. Nussenzweig

**Affiliations:** 1 Laboratory of Molecular Immunology, The Rockefeller University, New York, NY USA; 2 Humoral Immunology Unit, Institute Pasteur, Université Paris Cité, Paris, France; 3 Laboratory of Basic and Applied Virology, Microbiology Department, Institute of Biological Sciences, Federal University of Minas Gerais, Belo Horizonte, Brazil; 4 Laboratory of Antigen Targeting to Dendritic Cells, Parasitology Department, University of São Paulo, São Paulo, Brazil; 5 Center for Virology and Vaccine Research, Beth Israel Deaconess Medical Center, Harvard Medical School, Boston, MA USA; 6 Nuffield Department of Medicine, University of Oxford, Oxford, UK; 7 NIHR Oxford Biomedical Research Centre, University of Oxford, Oxford, UK; 8 School of Public Health, Imperial Clinical Trials Unit, Imperial College London, London, UK; 9 NIHR Imperial Biomedical Research Centre, Imperial College London, London, UK; 10 Department of Infectious Disease, Imperial College London, London, UK; 11 Guy’s and St Thomas Hospital NHS Foundation Trust, London, UK; 12 University College London, London, UK; 13 Central and Northwest London NHS Foundation Trust, London, UK; 14 University Hospitals Sussex NHS Foundation Trust, Brighton, UK; 15 Brighton & Sussex Medical School, Brighton, UK; 16 Manchester University NHS Foundation Trust, Manchester, UK; 17 Department of Clinical Medicine, Aarhus University Hospital, Aarhus, Denmark; 18 Western General Hospital, NHS Lothian Trust, Edinburgh, UK; 19 Chelsea and Westminster Hospital, London, UK; 20 Royal Free London NHS Foundation Trust, London, UK; 21 Queen Mary University of London, London, UK; 22 Barts Health NHS Trust, London, UK; 23 HIV i-Base, London, UK; 24 Department of Genetics, Ribeirao Preto Medical School, University of Sao Paulo, Ribeirão Preto, Brazil; 25 Howard Hughes Medical Institute, The Rockefeller University, New York, NY USA

**Keywords:** HIV infections, Randomized controlled trials

## Abstract

RIO is an ongoing phase 2 double-blind randomized placebo-controlled human trial. Sixty-eight adult men living with human immunodeficiency virus (HIV), who were predominantly white and initiated antiretroviral therapy (ART) during primary or early-stage infection, underwent treatment interruption and were randomly assigned to a group receiving one or two doses of two long-acting broadly neutralizing antibodies (bNAbs) 3BNC117-LS and 10-1074-LS (arm A, *n* = 34) or saline (arm B, *n* = 34). The primary clinical study met its primary endpoint and was published elsewhere, demonstrating significantly delayed viral rebound and prolonged ART-free control in participants receiving bNAbs compared with placebo. The infusions were generally safe and well tolerated, with no study-related serious adverse events reported. The most common reported treatment-related adverse events were fatigue, lethargy and somnolence. Here we report on prespecified secondary analyses examining viral rebound, antibody sensitivity and reservoir dynamics, as well as prespecified exploratory analyses of rebound virus evolution and autologous antibody activity. Preinfusion reservoir measurements showed low levels of intact proviral HIV-1 DNA in circulating CD4^+^ T cells in both arms. The rebounding viruses in participants who received the antibodies showed significant selection for resistance to 10-1074-LS but not to 3BNC117-LS. Notably, there was a significant correlation between greater initial reservoir sensitivity to autologous antibodies and 10-1074 and time to rebound. Finally, comparison of preinfusion and prerebound HIV-1 proviral reservoir showed a decrease in intact but not defective proviruses in arm A but not in arm B. Together, these findings suggest that baseline humoral immunity and prolonged exposure to LS-bNAbs jointly shape post-treatment viral rebound dynamics in individuals who initiate ART during primary infection. ClinicalTrials.gov identifier: NCT04319367.

## Abstract (web summary)

In the phase 2 RIO trial, there was delayed viral rebound and resistance to broadly neutralizing antibodies 3BNC117-LS and 10-1074-LS in adult males living with HIV undergoing analytical treatment interruption, and initial reservoir sensitivity to autologous antibodies was associated with a longer time to rebound.

## Main

Upon entry into CD4^+^ T cells, the human immunodeficiency virus (HIV-1) RNA genome is reverse transcribed and preferentially integrated into transcriptionally active sites in the host genome^1^. High-level transcription of replication-competent proviruses leads to cell death and, if untreated, almost invariably results in lethal immunodeficiency^2^. Antiretroviral therapy (ART) prevents the spread of infection and immunodeficiency but fails to eliminate a reservoir of latent proviruses found primarily in long-lived, resting memory CD4^+^ T cells^3–6^. These reservoir cells express viral RNA at levels below the threshold required to either induce cytopathic effects or elicit robust immune-mediated clearance, yet they can retain the capacity to reinitiate systemic infection upon ART interruption^7–9^.

The HIV-1 reservoir contains both intact and defective proviruses and has been characterized by several different methods including viral outgrowth assays, digital droplet polymerase chain reaction (ddPCR) and whole-genome amplification and sequencing (quadruplex four-probe PCR (Q4PCR))^10–13^. Whereas the size of the defective reservoir is relatively stable over time on ART, the intact reservoir shows early, rapid decay that slows to a half-life of approximately 4.5 years, with possible further prolongation after 7 years on therapy^14–17^. Intact proviruses remaining in the reservoir after long-term therapy tend to reside in areas of the genome that are less transcriptionally active^18–20^. Although the mechanism of selection against the reservoir viruses integrated into transcriptionally active parts of the genome is not precisely understood, selection is thought to be mediated by a combination of HIV-1 cytopathic effects and host immune responses.

Passively administered antibodies can enhance host immune responses and induce simian HIV control by a CD8^+^ T cell dependent mechanism^21–23^. In addition, several small studies in humans have reported that broadly neutralizing antibody (bNAb) therapy is associated with accelerated clearance of infected cells, delayed viral rebound and a higher-than-expected rate of prolonged control^24–29^. Control has been associated with increased HIV-1–specific CD8^+^ T cell responses in some but not all studies^29–32^. In addition, observational studies in post-treatment controllers, people living with HIV (PLWH) and tissue culture experiments suggest that autologous antibodies may also put pressure on the reservoir^33–36^. Together, these findings provided the rationale for evaluating whether prolonged exposure to long-acting bNAbs could enhance post-treatment viral control and for investigating the virologic and immunologic correlates associated with this outcome.

The RIO trial is a randomized, double-blind, placebo-controlled phase 2 study evaluating the long-acting bNAbs 3BNC117-LS and 10-1074-LS administered during analytical treatment interruption (ATI) in individuals who initiated ART during primary or early HIV infection^37,38^. Here we report on prespecified secondary and exploratory analyses including viral reservoir composition and antibody sensitivity that shape rebound dynamics and post-treatment control in the RIO trial.

## Results

### Trial overview

**Extended Data Fig. 1:**
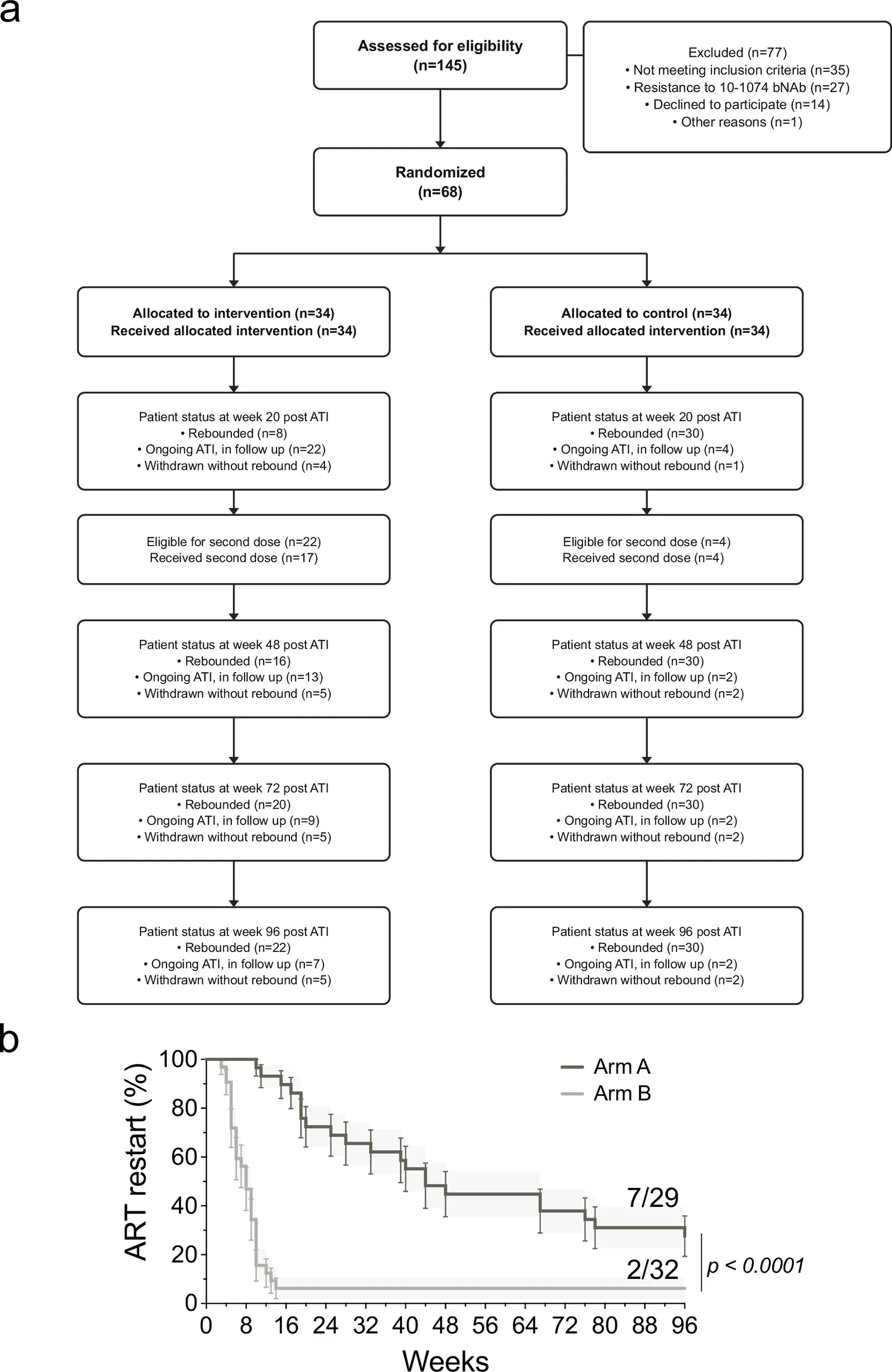
Trial participant selection and time to ART restart after ATI. **a**, CONSORT flow diagram showing participant enrollment, randomization, allocation to Arm A or Arm B, follow-up, ART restart categories, and inclusion in virologic analyses. **b**, Kaplan–Meier curves showing time to ART restart (up to 96 weeks) in participants from Arm A (bNAbs; *n* = 29) and Arm B (placebo; *n* = 32). Shaded areas indicate the standard error (SE) of the survival estimate. Two-sided Gehan–Breslow–Wilcoxon test, χ^2^ = 30.09, P <0.0001. Numbers at the end of each curve indicate the number of participants remaining off ART at week 96 relative to the total in each arm.

Between 17 May 2021 and 30 July 2024, the RIO trial enrolled 68 participants who were randomized 1:1 to receive two bNAbs, 3BNC117-LS and 10-1074-LS (arm A) or placebo (arm B) 2 days before ATI. Baseline demographic and clinical characteristics were balanced between the two study arms. All participants were men, with a median age of 40 years, and most identified as white (58/68, 85.3%) (Extended Data Table 1). The primary outcome, reported elsewhere^38^, was time to viral rebound within 20 weeks after ATI. Prespecified secondary outcomes were safety, delayed viral rebound and prolonged ART-free viral control after ATI. Additional prespecified secondary outcomes, including CD4^+^ T cell recovery, pharmacokinetic analyses, antidrug antibody responses and quality-of-life assessments, have also been reported in the primary clinical report^38^. The infusions were generally safe. ART was restarted if the viral load (VL) was >100,000 HIV-1 copies per ml for 2 consecutive weeks or >1,000 HIV-1 copies per ml for 6 weeks or CD4^+^ T cell count <350 cells per ml or clinical symptoms or participant preference^38^. Early rebound before week 20 occurred in 8 arm A and 30 arm B participants: 75% versus 11% (*P* < 0.001; Extended Data Fig. 1). The median time to ART restart was 45.4 weeks in arm A, where 7 of 29 participants (24%) remained off ART at 96 weeks, compared with 4.6 weeks in arm B, where only 2 of 32 (6%) maintained undetectable HIV-1 levels throughout 96 weeks of observation (*P* < 0.001; Extended Data Fig. 1)^38^. Notably, 13 of the 29 individuals in arm A restarted ART before meeting virological restart criteria^38^.

Prespecified secondary outcomes presented in this study include quantitation of viral reservoir size and dynamics, time to ART restart and reservoir viral sensitivity and resistance to the administered bNAbs. Prespecified exploratory outcomes include rebound virus sequencing, phylogenetic analyses, reservoir characterization and virologic correlates of post-treatment viral control. Additional prespecified exploratory investigations, including immune phenotyping, host gene expression and HIV integration site analyses, are beyond the scope of the present study and will be reported separately. Analyses of autologous antibody activity were not prespecified in the protocol or Statistical Analysis Plan and are presented here as non-prespecified exploratory analyses. Post hoc analyses were limited to subgroup analyses of participants with distinct rebound patterns following bNAb therapy.

### Baseline reservoir

To examine the HIV-1 proviral reservoir in individuals enrolled in the RIO trial, we assayed preintervention peripheral blood mononuclear cells (PBMCs). Reservoir size was estimated by ddPCR and Q4PCR^10,11^. ddPCR documents the presence of two conserved regions in the provirus: the packaging signal and the envelope^10^. It is more sensitive, less expensive and scalable than Q4PCR^11^. However, it does not allow sequence confirmation, and therefore some of the proviruses that are assigned as intact are in fact defective, a problem that can increase with time because the intact reservoirs decay more quickly than the defective ones^39^. Q4PCR provides sequence information and direct confirmation of whether a provirus is intact or defective, but it is less sensitive than ddPCR because it requires amplification of the nine-kilobase proviral genome^40^.

**Extended Data Fig. 2:**
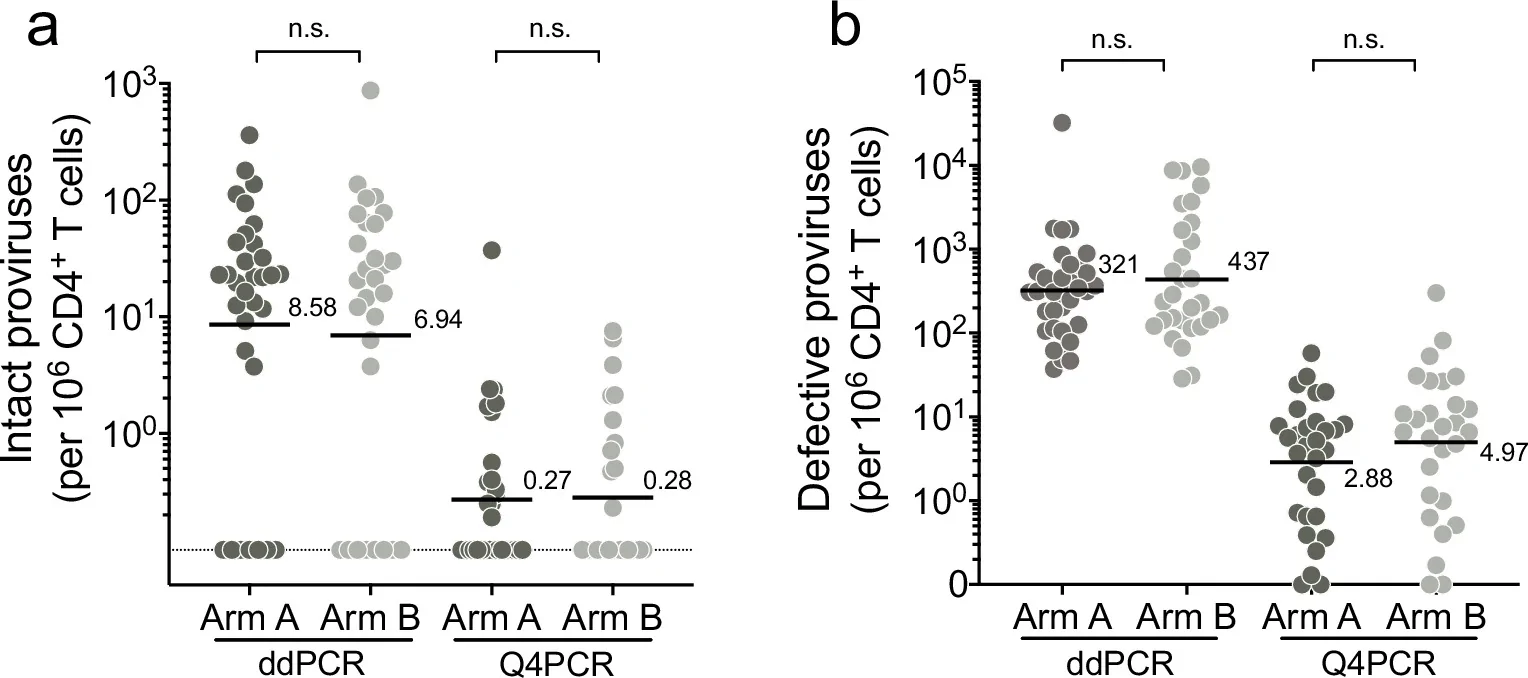
Baseline HIV reservoir in circulating CD4+ T cells measured by ddPCR and Q4PCR. Graphs show the numbers of (**a**) intact and (**b**) defective proviral genomes per million CD4+ T cells. Each dot represents the value for one participant. Arm A participants black and Arm B grey. Sample size: ddPCR – Arm A, n = 32; Arm B, n = 29; Q4PCR – Arm A, n = 30; Arm B, n = 27. The black line and numbers indicate the geometric mean. Group comparisons were performed using a two-sided Mann–Whitney test, with statistical significance defined as P <0.05.

To examine the preintervention reservoir in the RIO participants, we performed both assays. Individuals in the two arms (ddPCR: arm A *n* = 32, arm B *n* = 29; Q4PCR: arm A *n* = 30, arm B *n* = 28) showed similar numbers of intact and defective proviruses in ddPCR and Q4PCR assays (Extended Data Fig. 2a,b and Supplementary Table 1). The geometric mean number of intact proviruses per million CD4^+^ T cells was 8.58 and 6.94 by ddPCR (*P* = 0.5) and 0.27 and 0.28 by Q4PCR (*P* = 0.5) for arms A and B, respectively (Extended Data Fig. 2a). The relatively small size of the reservoir in this cohort is consistent with early therapy^41–43^.

**Fig. 1:**
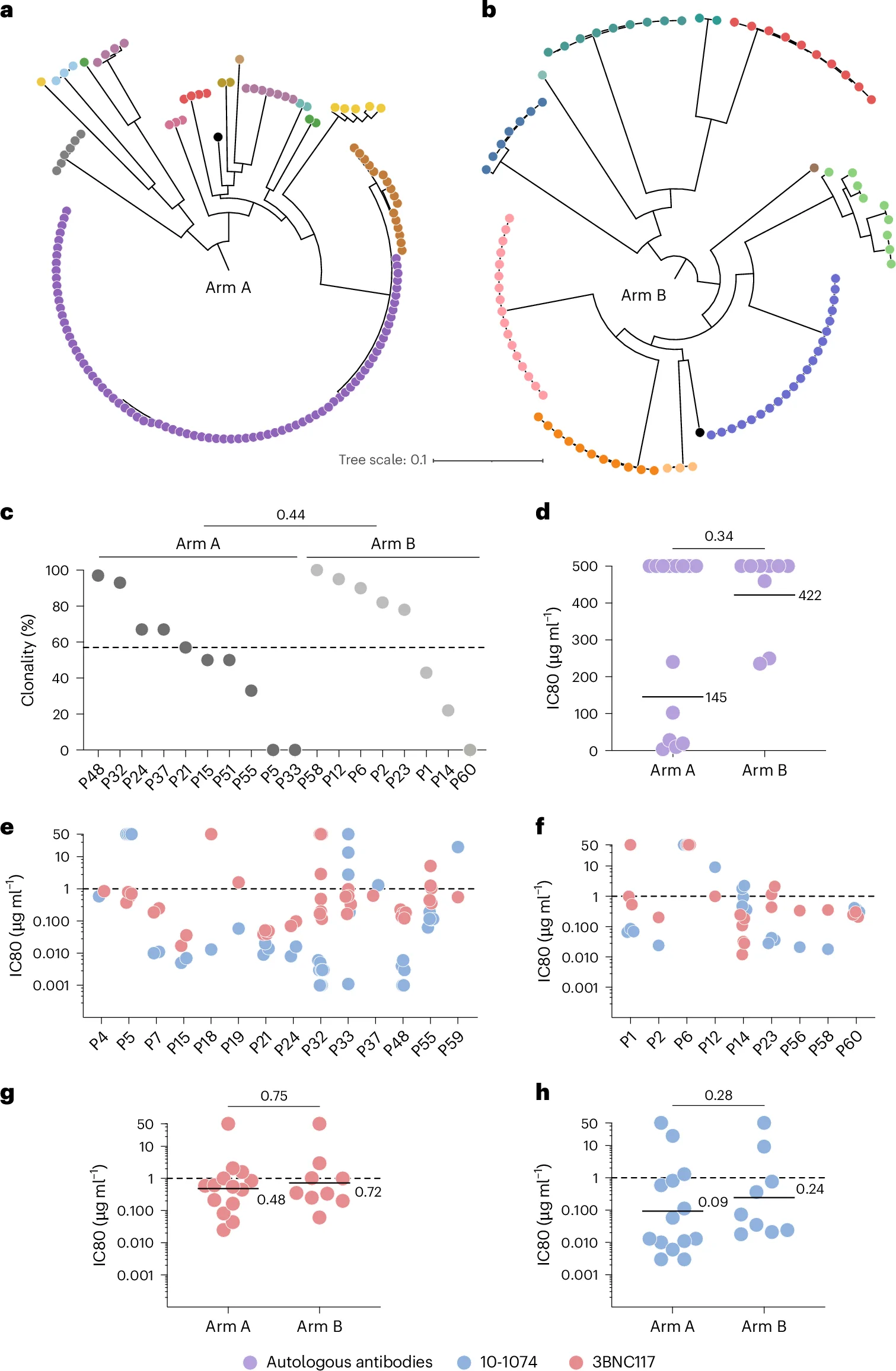
Baseline reservoir characteristics. **a**,**b**, Circular phylogenetic trees using the generalized time-reversible (GTR) model depict clonal distribution of intact proviral nucleotide sequences based on *env* in arm A (**a**) and arm B (**b**). Colors represent different participants. Black dot is HXB2. **c**, Intact reservoir clonality for the indicated participants, calculated based on amino acid sequences of *env* for individuals with ≥3 intact proviral sequences. Dashed line indicates combined average clonality of both arms. **d**, Baseline reservoir sensitivity to preinfusion autologous IgG antibodies, expressed as IC80. Each dot represents one participant (one biological replicate), using the geometric mean IC80 of all reservoir-derived pseudoviruses from that participant. Lines and numbers show overall geometric mean for all participants in arms A (*n* = 13) and B (*n* = 9). **e**,**f**, Baseline reservoir neutralization sensitivities to 3BNC117 (red) and 10-1074 (blue), expressed as IC80, for individual pseudovirus IC80s for the indicated participants in arm A (**e**) and arm B (**f**). Dotted lines indicate 1 mg ml^−1^. **g**,**h**, Each dot represents one participant (one biological replicate), using the geometric mean IC80 of all reservoir-derived pseudoviruses from that participant against 3BNC117 (**g**) and 10-1074 (**h**). Arm A *n* = 14 and arm B *n* = 9. Group comparisons in **c**,**d** and **g**,**h** were performed using a two-sided Mann–Whitney test, with statistical significance defined as *P* < 0.05.

We were able to recover 141 and 91 intact proviral reservoir sequences from baseline samples from 15 and 11 individuals in arms A and B, respectively (Fig. 1a,b and Supplementary Table 2). We were unable to recover intact proviruses from the remaining participants due to their small reservoirs. As expected, each participant harbored a separate non-overlapping group of intact HIV-1 proviral sequences (Fig. 1a,b). Despite early therapy and the small reservoir sizes, intact proviruses showed a median level of clonality of 57% when considering all individuals from whom we were able to obtain three or more intact proviral sequences (*n* = 10 for arm A and *n* = 8 for arm B)^12,44–46^ (Fig. 1c and Supplementary Table 2).

**Extended Data Fig. 3:**
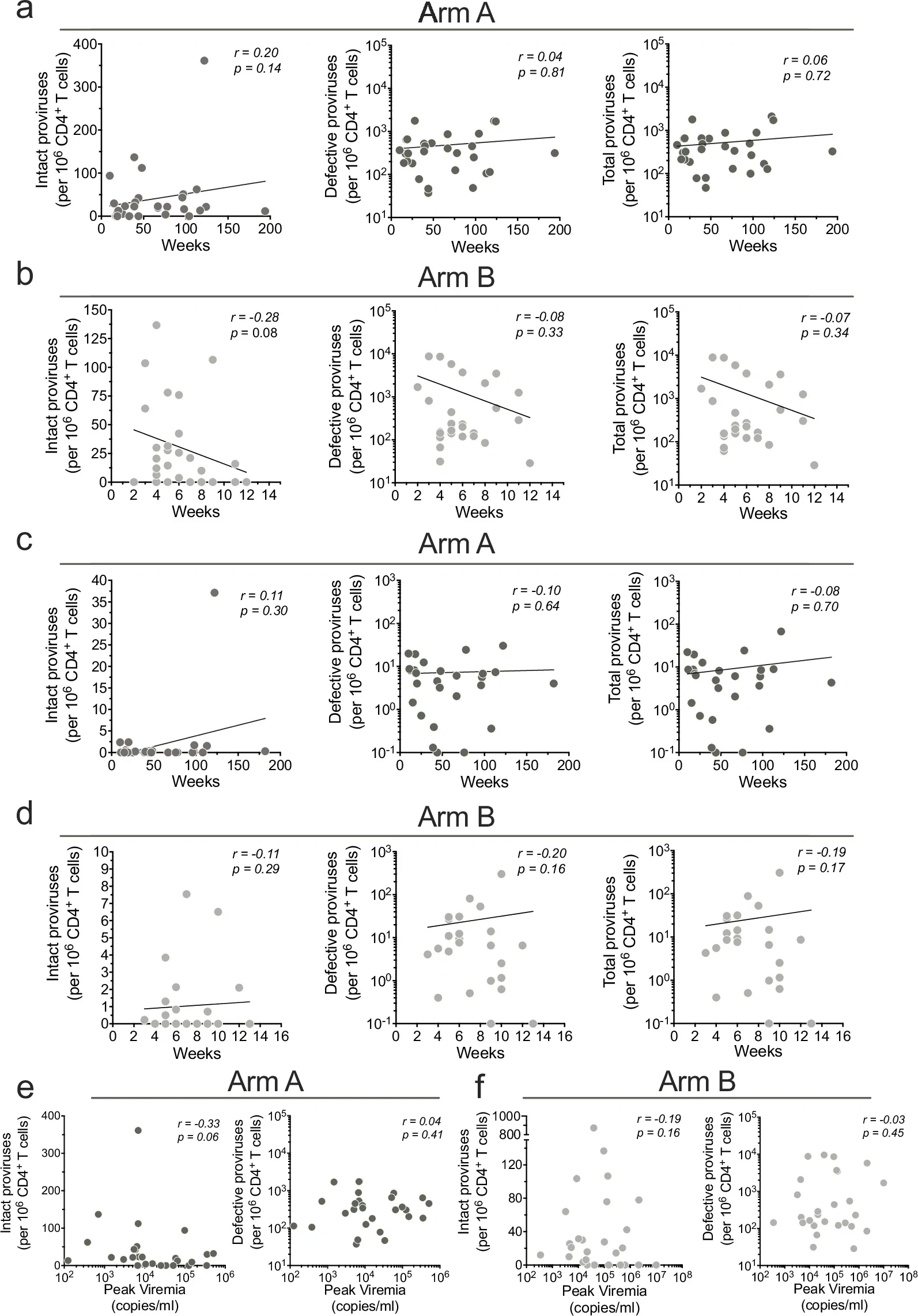
Correlation between baseline intact, defective and total reservoir size, peak viremia and time to ART restart in weeks. (**a** - **d**) Correlation between baseline intact, defective, and total proviral reservoir size measured by ddPCR (**a**-**b**, **e**-**f**) or Q4PCR (**c**,**d**) and time to ART restart in weeks in Arm A (**a**,**c**) and Arm B (**b**,**d**). Each dot represents one participant. For ddPCR measurements, n = 29 for Arm A and n = 27 for Arm B. For Q4PCR measurements, n = 26 for Arm A and n = 25 for Arm B. (**e**,**f**) Relationship between baseline reservoir size measured by ddPCR and peak plasma viremia in Arm A (**e**) and Arm B (**f**). Each dot represents one participant. n = 28 for Arm A and n = 27 for Arm B. Y axes indicate the number of intact or defective proviruses, and x axes indicate plasma viremia at peak rebound. Correlation coefficients were calculated using a two-sided Spearman’s rank correlation with 95% confidence intervals. Nonlinear curve fitting was performed with x as a linear function, and y as a linear or logarithmic function for intact and defective genomes, respectively.

To determine whether baseline reservoir size was associated with virologic outcomes after ATI, we compared intact proviral reservoir measurements with time to ART restart and peak viremia. There was no significant correlation between the size of the intact proviral reservoir and time to ART restart in the placebo or bNAb arm of the study, as measured by either ddPCR or Q4PCR (Extended Data Fig. 3). In addition, there was no correlation between the reservoir size and peak viremia before ART restart in either group (Extended Data Fig. 3).

**Extended Data Fig. 4:**
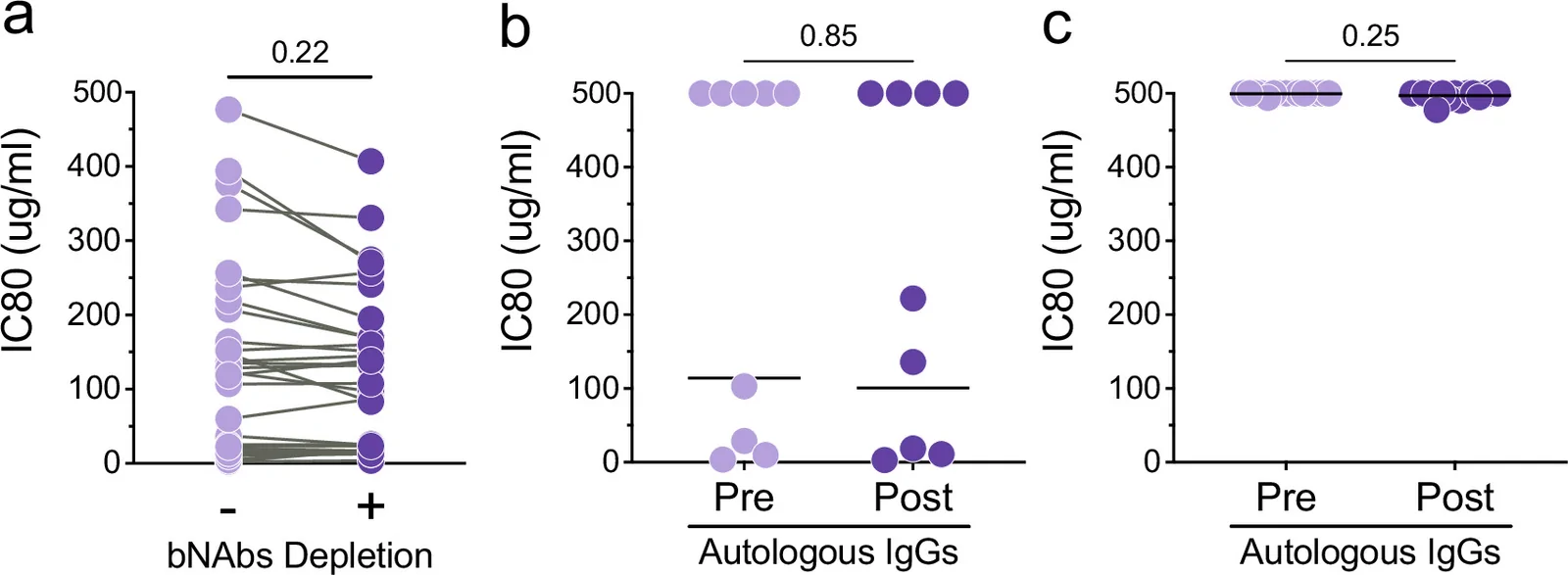
Autologous neutralization activity and breadth post-bNAb therapy in Arm A participants. (**a**) Control depletion experiments performed in six individuals comparing depleted and non-depleted IgG preparations against participant-matched autologous viruses demonstrated that the depletion procedure preserved endogenous autologous neutralizing activity. (**b**) Autologous neutralization sensitivity (IC80) of baseline reservoir-derived pseudoviruses tested against IgG isolated before bNAb infusion and at later post-infusion time points after depletion of residual infused bNAbs. Each circle represents the geometric mean IC80 for one participant (one biological replicate, n = 9). Post-infusion IgG samples were collected at the following time points, with corresponding residual plasma concentration (µg ml-1) of 3BNC117 and 10-1074, respectively: P4, week 39 (12.5 and 10.7); P7, week 64 (4.1 and 4.7); P15, week 134 (<1 and <1); P18, week 64 (13.8 and 18.1); P19, week 36 (25.9 and 30.0); P21, week 119 (2.2 and 3.3); P32, week 123 (<3 and <3); P33, week 27 (338 and 32.5); and P48, week 56 (22.0 and 20.8). (**c**) Neutralization breadth of autologous IgG isolated before and after bNAb therapy was evaluated in a panel of 10 HIV-1 strains resistant to both 3BNC117 and 10-1074. Analyses included 15 participants, with post-infusion IgG samples collected at a mean of 65.7 weeks after infusion. Group comparisons were performed using a two-sided Wilcoxon matched-pairs signed-rank test (P <0.05).

Sixty-five pseudotype viruses from distinct intact reservoir-derived *env* sequences from 14 and 9 individuals in arms A and B, respectively, were tested for neutralization sensitivity to autologous baseline IgG, 3BNC117 and 10-1074 in TZM-bl assays^47^ (Fig. 1d–h and Supplementary Tables 2 and 4). Analysis of autologous antibody responses was performed as a non-prespecified exploratory analysis. Consistent with early therapeutic intervention, only 8 of these 23 individuals showed autologous antibody neutralizing activity, with an 80% inhibitory concentration (IC80) of <250 µg ml^−1^ of purified IgGs (corresponding to ≅1:100 dilution of serum) against their reservoir proviruses (Fig. 1d and Supplementary Tables 2 and 4). To assess whether autologous neutralizing activity changed over time, we performed longitudinal neutralization assays following depletion of residual infused bNAbs (Methods). Control experiments demonstrated that the depletion procedure efficiently removed therapeutic bNAbs while preserving autologous neutralizing activity (Extended Data Fig. 4a). Autologous IgG was tested both against baseline reservoir-derived viruses and against a panel of HIV strains resistant to 3BNC117 and 10-1074 to evaluate neutralization breadth. We found no increase in autologous neutralizing activity against baseline reservoir viruses and no measurable expansion of neutralization breadth in samples collected at a mean of 68 weeks after ATI from individuals in arm A (Extended Data Fig. 4).

Reservoir sensitivity to each of the two bNAbs ranged from an IC80 of <0.001 to >50 µg ml^−1^ for 10-1074 and 0.012 to >50 µg ml^−1^ for 3BNC117, with no significant differences between the two study arms (Fig. 1e–h). The geometric mean IC80s were 0.48 and 0.72 µg ml^−1^ for 3BNC117 and 0.09 and 0.24 for 10-1074 for arms A and B, respectively (Fig. 1g,h and Supplementary Table 4). All individuals in the bNAb arm showed reservoir proviral sensitivity to at least one of the two bNAbs (Supplementary Tables 2 and 4).

### Rebound viremia

**Fig. 2:**
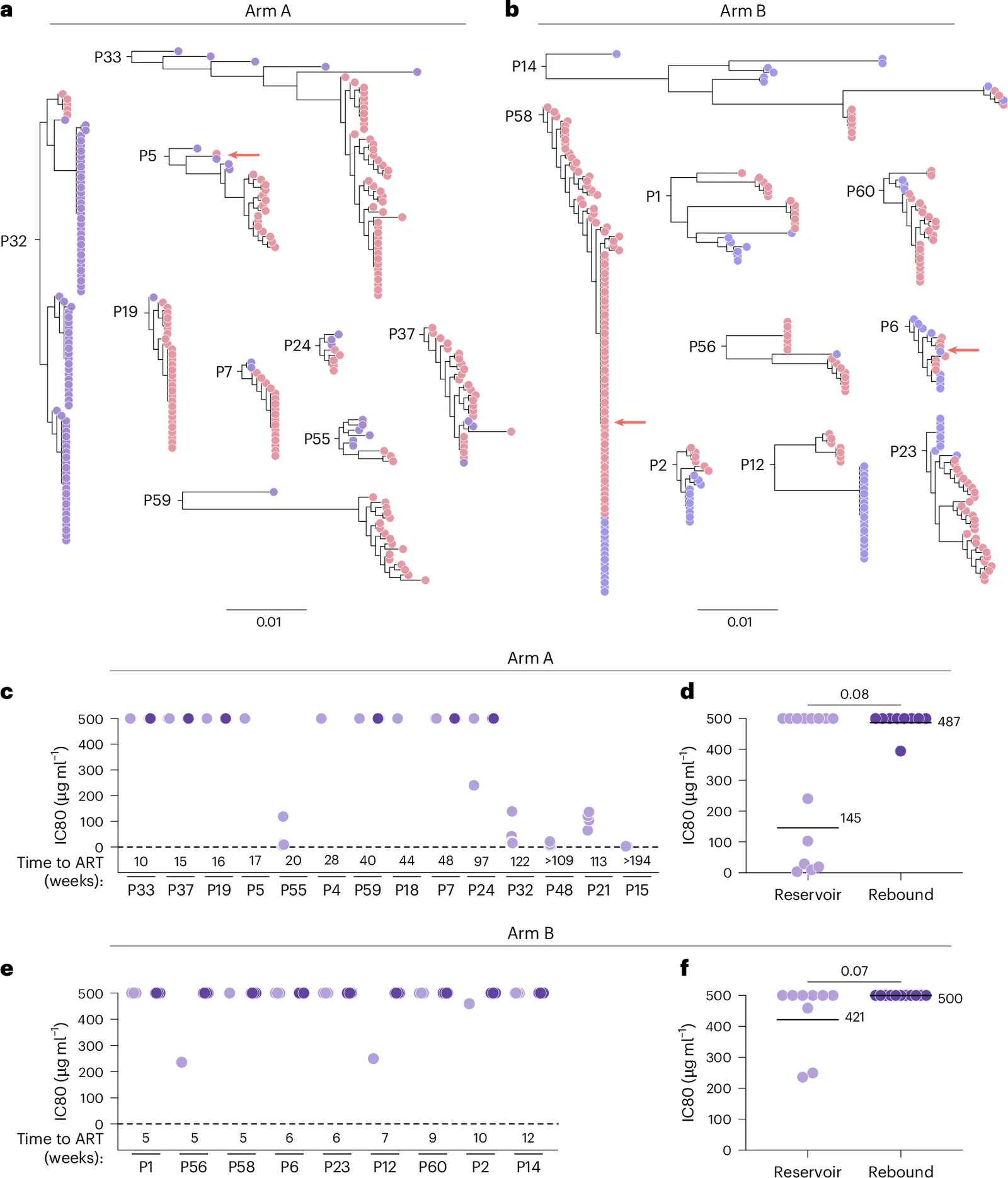
Relationship between intact proviral reservoir and rebound *env* nucleotide sequences from participants in both study arms. **a**,**b**, Phylogenetic trees were constructed using the GTR model for participants in arm A (**a**) and arm B (**b**). Intact reservoir proviruses are shown in purple and rebound in red. Red arrows indicate sequences shared between reservoir and rebound. **c**–**f**, Neutralization of reservoir and rebound pseudoviruses by autologous antibodies. Individual pseudovirus IC80s for participants from arm A (**c**) and arm B (**e**). Numbers at the bottom indicate the time to ART restart in weeks. Each dot represents one participant (one biological replicate) from arm A (**d**) or arm B (**f**), using the geometric mean IC80 of all pseudoviruses from that participant. Arm A reservoir *n* = 13 and rebound *n* = 9. Arm B reservoir *n* = 9 and rebound *n* = 11. Lines and numbers indicate geometric mean for all participants. Group comparisons in **d** and **f** were performed using a two-sided Mann–Whitney test, with statistical significance defined as *P* < 0.05.

**Fig. 3:**
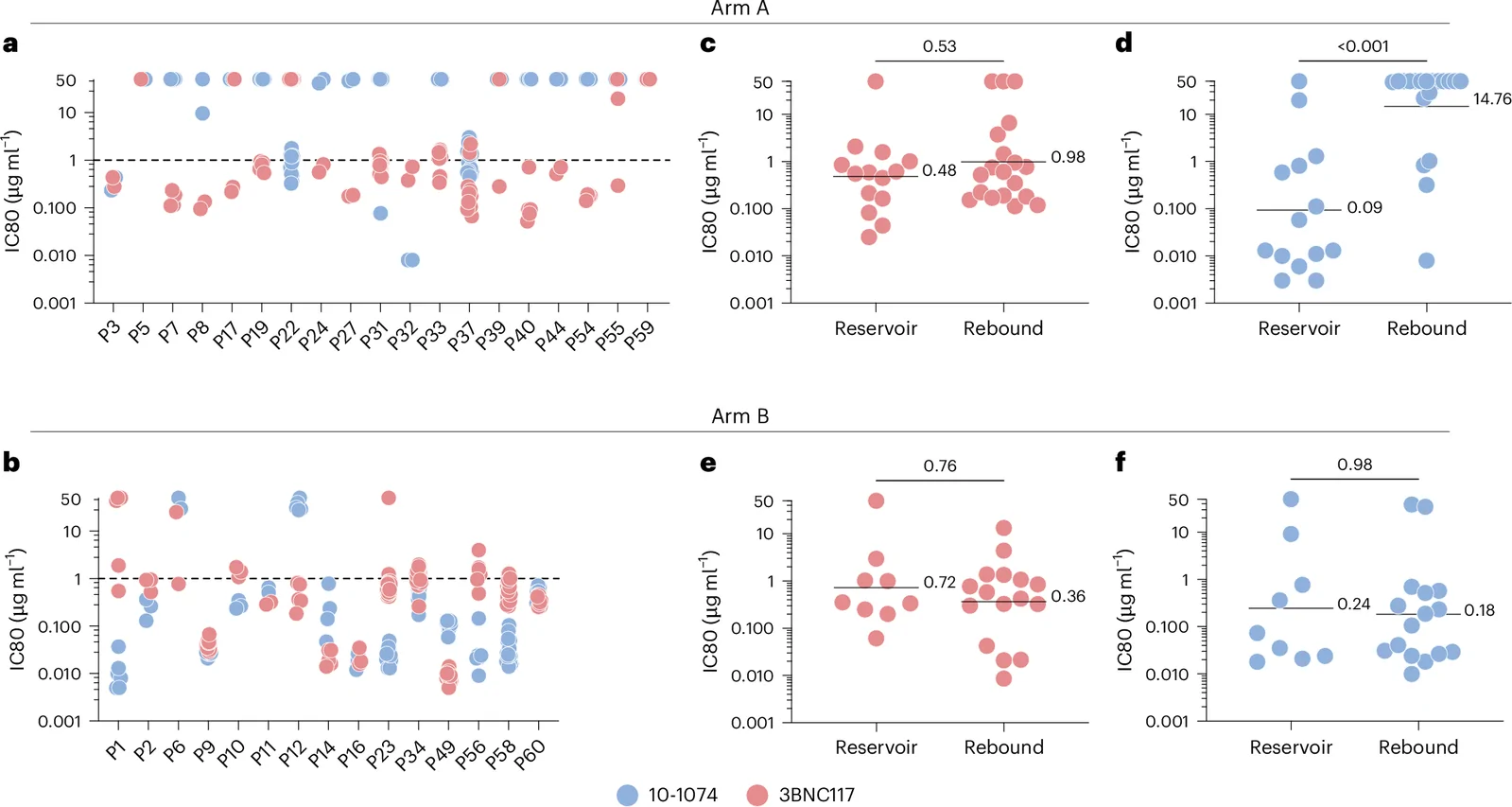
Pseudovirus neutralization. Pseudovirus neutralization by 3BNC117 (red) and 10-1074 (blue) for participants expressed as IC80. **a**,**b**, Each dot represents an individual rebound pseudovirus for arm A (**a**) or arm B (**b**). **c**–**f**, Each dot represents the geometric mean IC80 for reservoir and rebound pseudoviruses in one participant (one biological replicate). Arm A, 3BNC117 (**c**) and 10-1074 (**d**) (reservoir *n* = 14, rebound *n* = 19); arm B, 3BNC117 (**e**) and 10-1074 (**f**) (reservoir *n* = 9, rebound *n* = 16). Line and numbers indicate geometric mean for all participants. Group comparisons in **c**,**d** and **e**,**f** were performed using a two-sided Mann–Whitney test, with statistical significance defined as *P* < 0.05.

We were able to obtain 323 and 370 rebound viral *env* sequences by single-genome amplification from 19 and 16 individuals who experienced viral rebound in arms A and B, respectively (Supplementary Table 3). To examine the nature of rebound viremia and its relationship to the proviral reservoir, we compared reservoir and rebound *env* sequences from 18 individuals from whom we were able to obtain both intact reservoir and rebound sequences (Fig. 2a,b). Pseudotype viruses obtained from participants in arms A (96) and B (119) were produced and tested for sensitivity to the two bNAbs and to autologous antibodies obtained at the time of entry (Figs. 2c–f and 3a–f and Supplementary Table 3).

Rebound virus sequences rarely overlap with the dominant latent intact proviral clones found in the circulating reservoir^48,49^. This phenomenon remains poorly understood but has been attributed in part to suppression of reservoir viruses by autologous antibodies^35,50^. Among 33 intact latent proviral reservoir clones (91 sequences) obtained from 9 control-arm participants, we found only 2 that overlapped with rebound sequences (Fig. 2b). There was no overlap between the reservoir and rebound sequences in the remaining seven control participants, only three of whom had measurable levels of autologous neutralizing antibodies to the reservoir (Fig. 2e and Supplementary Tables 2 and 4). Thus, autologous antibody neutralizing activity cannot entirely account for the disparity between intact proviruses found in the reservoir and the dominant viruses found at the time of rebound in this study of individuals receiving early ART therapy. Nevertheless, rebound viruses in the three individuals from the control group who had measurable autologous neutralizing antibodies to the reservoir were resistant to their own antibodies (Fig. 2d–f). As expected, when autologous antibodies are present at baseline (during ART), rebound viruses are enriched in variants that are autologous antibody resistant^33,34^.

Pseudotype viruses obtained from rebound were also tested for sensitivity to the two bNAbs (Fig. 3). Consistent with the absence of selective pressure in the control arm, the geometric mean bNAb sensitivity of all reservoir and rebound pseudotype viruses obtained from arm B participants was not significantly different for either antibody (3BNC117 *P* = 0.76, 10-1074 *P* = 0.98; Fig. 3b,e,f).

**Extended Data Fig. 5:**
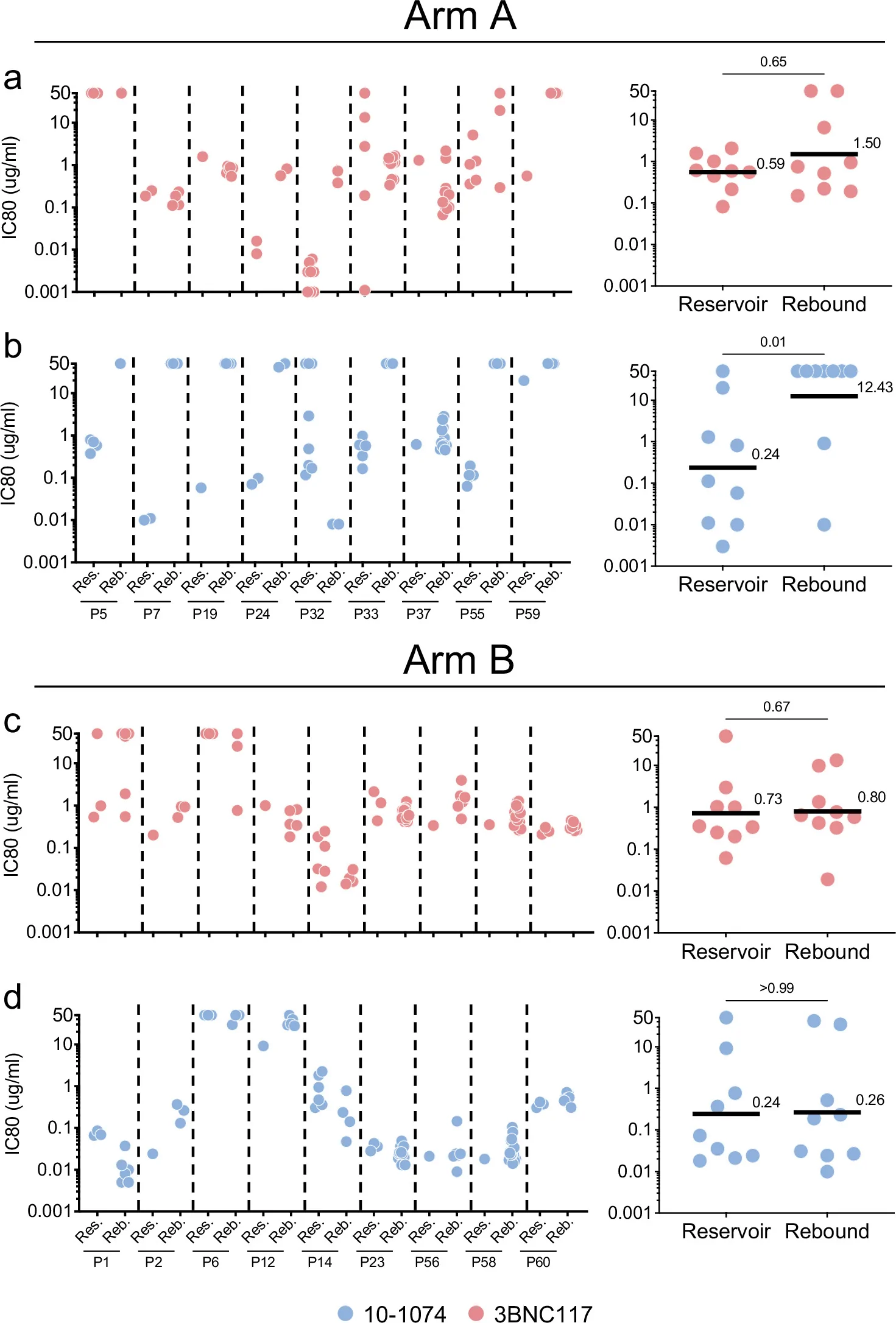
Per-participant matched reservoir- and rebound-derived pseudovirus neutralization sensitivities to 3BNC117 and 10-1074 in both study arms, expressed as IC80 values. **a**-**d**, Each dot represents a reservoir- or rebound-provirus from each participant in (**a**, **b**) Arm A and (**c**, **d**) Arm B. N = 27 participants for each Arm. Members of expanded clones are represented only once, irrespective of the size of the clone. Geometric mean IC80 values of all reservoir- and rebound-derived clones per participant are indicated alongside each graph and were used for statistical comparison. Group comparisons were performed using a two-sided Mann–Whitney test, with statistical significance defined as P <0.05. Black lines indicate the geometric mean.

In contrast, selection for bNAb resistance was evident among rebound viruses obtained from arm A participants (Fig. 3a,c,d). The geometric mean neutralizing IC80s for 10-1074 against reservoir and rebound pseudoviruses was 0.09 and 14.7 mg ml^−1^, respectively, with the majority of the rebound viruses being completely resistant (IC80 > 50 µg ml^−1^) (*P* < 0.001; Fig. 3d and Extended Data Fig. 5b). In contrast, there was a much smaller difference in the geometric mean IC80s between reservoir and rebound for 3BNC117 that did not reach statistical significance (*P* = 0.53; Fig. 3c and Extended Data Fig. 5a). Only 3 of 19 participants who received bNAbs showed rebound viruses that were uniformly resistant to 3BNC117 (Fig. 3a and Supplementary Tables 3 and 4). In conclusion, 10-1074 escape variants were far more likely to emerge than 3BNC117 resistant variants during dual bNAb therapy, as reported in earlier studies^25,27,51^.

### Viral control and reservoir antibody sensitivity

**Fig. 4:**
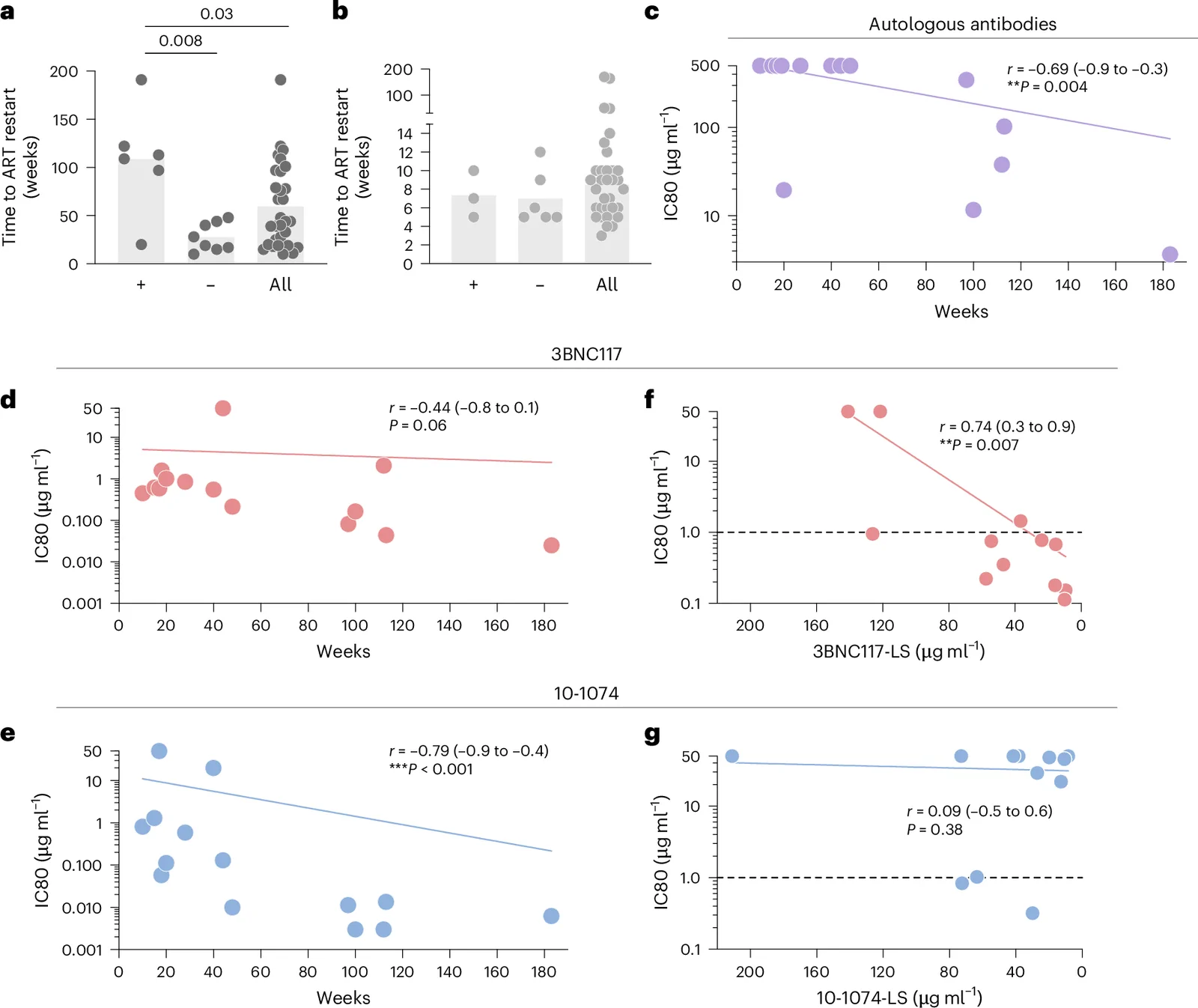
Viral suppression and reservoir sensitivity to autologous antibodies, 3BNC117 and 10-1074. **a**,**b**, Time to ART restart for individuals who did (+) or did not (−) display reservoir sensitivity to autologous antibodies or all participants; arm A (**a**) and arm B (**b**). Each dot represents the time to rebound for an individual participant (one biological replicate). Autologous antibody analyses included arm A: *n* = 6 (+) and *n* = 8 (−); arm B: *n* = 3 (+) and *n* = 6 (−). The ‘all participants’ groups include all randomized participants (arm A, *n* = 30; arm B, *n* = 34). The bars represent the geometric mean for each group. Two-sided Mann–Whitney group comparisons, with *P < 0.05, **P < 0.01, ***P < 0.001 considered significant. **c**–**e**, Correlation between time to ART restart and baseline reservoir sensitivity to autologous antibodies (**c**), 3BNC117 (**d**) and 10-1074 (**e**). Each dot represents the geometric mean IC80 of all reservoir-derived pseudoviruses from one participant (one biological replicate). Correlations were performed using one value per participant (*n* = 14). **f**,**g**, Correlation between IC80 from rebounding viruses for 3BNC117 (**f**) or 10-1074 (**g**) and the bNAb concentration at the time of rebound. Each dot represents one participant (one biological replicate), using the geometric mean IC80 (*y* axis) of all rebound-derived pseudoviruses from that participant and the corresponding serum bNAb concentration (*x* axis) at rebound (*n* = 12). Nonlinear regression was performed using a linear *x* axis and log-transformed *y* axis. Correlation coefficients were calculated using two-sided Spearman’s *r* with 95% confidence intervals.

**Extended Data Fig. 6:**
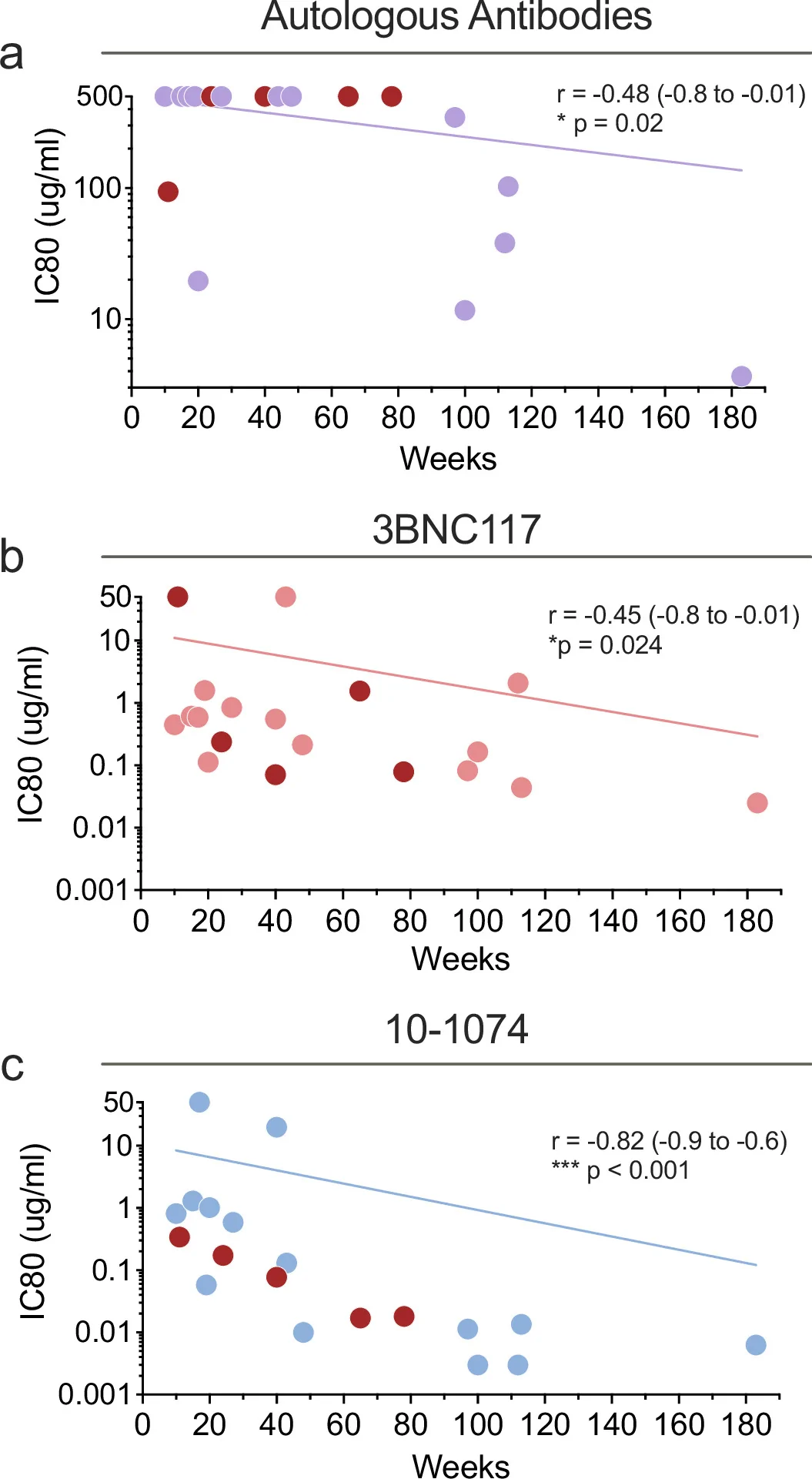
Reservoir sensitivity to autologous antibodies, 3BNC117 and 10-1074 including participants with intact *env* but not confirmed intact proviral genomes. **a**-**c**, Correlation between time to ART restart and baseline reservoir sensitivity to (**a**) autologous antibodies, (**b**) 3BNC117, and (**c**) 10-1074. Each dot represents one participant (one biological replicate), using the geometric mean IC80 (Y axis) and time to rebound in weeks (X axis) for one participant. Red dots represent participants that we were able to detect intact envs but not confirmed intact provirus. Nonlinear curve fitting was performed with x as a linear function of time and y as a logarithmic function of IC80 values. Correlation coefficients were calculated using a two-sided Spearman’s r with 95% confidence intervals.

**Extended Data Fig. 7:**
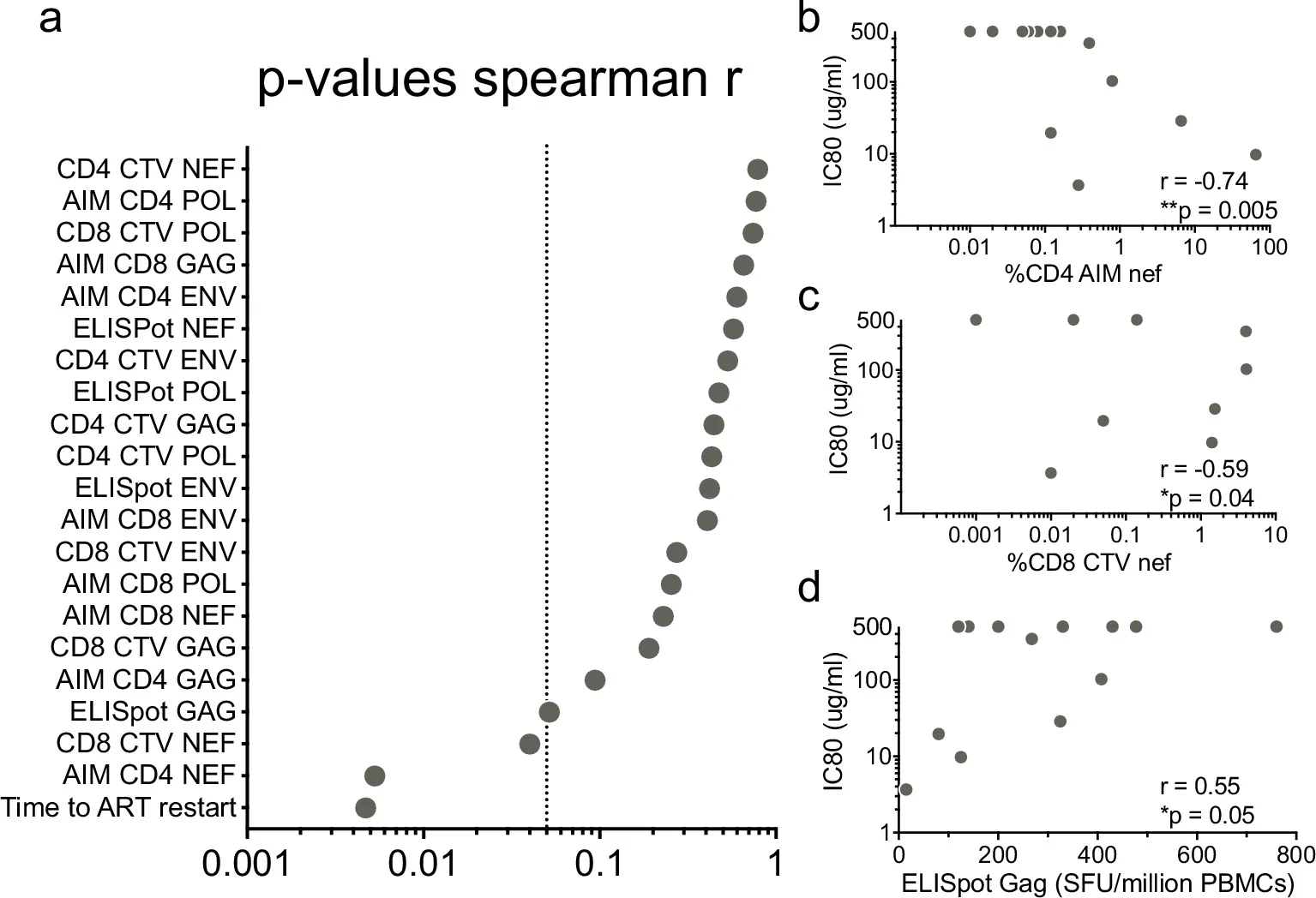
Associations between baseline autologous neutralizing activity and cellular immune responses. (**a**) Summary of two-sided Spearman correlations between baseline autologous neutralizing activity against reservoir viruses and cellular immune responses measured by ELISpot, CTV, and AIM assays at week 0. P values are indicated for each assay; the dashed line marks P = 0.05. (**b**–**d**), Scatter plots showing correlations between autologous antibody neutralization (IC_80_) and (**b**) AIM CD4^+^ responses to Nef, (**c**) CD8^+^ CTV responses to Nef, and (**d**) ELISpot responses to Gag peptides. Each point represents one participant (one biological replicate).

To determine whether reservoir sensitivity to either autologous antibodies or bNAbs was associated with control of viremia, we compared baseline autologous neutralizing titers and bNAb IC80s to reservoir viruses and time of ART restart (Fig. 4a–g). These analyses were limited to participants from whom reservoir viral sequences could be obtained. The mean time to ART restart among the three individuals in the control group who exhibited autologous antibody neutralizing activity against reservoir viruses was 7.3 compared with 8.5 weeks to all participants (Fig. 4b). Thus, in this small sample we see no measurable effect of the autologous antibodies in the absence of bNAb therapy. In contrast, the mean time to ART restart in the six bNAb recipients who showed baseline autologous neutralizing activity was 108 weeks compared with 27.5 to the 13 who did not (*P* = 0.008; Fig. 4a). Consistent with this observation, there was a significant correlation between preinfusion autologous neutralizing antibody titers to reservoir viruses and time to ART restart for individuals in the bNAb arm (*n* = 14, *r* = −0.69, *P* = 0.004; Fig. 4c). Time to ART restart was also correlated with initial reservoir proviral sensitivity to 10-1074 (*r* = −0.79, *P* < 0.001), but there was a trend only for 3BNC117 (*r* = −0.45, *P* = 0.06; Fig. 4d,e). Although we were unable to obtain additional intact proviral reservoir sequences, we were able to obtain intact *envs*, which were used to construct pseudotype viruses that were tested for sensitivity (*n* = 5, arm A) (Supplementary Table 2). When combined with the data obtained from intact proviruses, the correlation with time to ART restart remained significant for 10-1074 and autologous antibodies and became significant for 3BNC117 (Extended Data Fig. 6). There were no consistent associations between baseline autologous antibodies and baseline HIV-specific CD4^+^ or CD8^+^ T cell responses^52^ (Extended Data Fig. 7). Although correlations were observed for individual assays (AIM CD4 response to Nef and CD8 CTV response to Nef), these were not consistent across assays and sample size was limited precluding robust comparisons (Extended Data Fig. 7).

Although potentially insufficient on their own, persistent low levels of the two long-acting antibodies could contribute to control. The mean concentration of 3BNC117-LS and 10-1074-LS at 96–123 weeks after bNAb infusion in seven arm A controllers was 0.70 and 0.96 µg ml^−1^, respectively (Supplementary Table 5). To evaluate the combined influence of endogenous and therapeutic antibody pressure on rebound in arm A participants, we performed time-to-event analyses, modeling time to ART restart as a function of baseline reservoir sensitivity to autologous antibodies, 10-1074 and 3BNC117 using IC80 values. In univariable analyses, resistance to both autologous antibodies and 10-1074 was associated with shorter time to ART restart. Because reservoir sensitivity to autologous antibodies tended to positively correlate with sensitivity to 10-1074 (and to a lesser extend 3BNC117), these variables were partially collinear and may reflect overlapping antiviral susceptibility profiles. Consistent with this, the associations were attenuated in exploratory multivariable models when included together (Supplementary Table 6). Notably, autologous antibodies and 10-1074 retained significance when adjusted for 3BNC117 alone.

There was an inverse correlation between 3BNC117 concentration at the time of rebound and the IC80 of the rebounding virus to the bNAb (Fig. 4f). In contrast, this relationship was not observed for 10-1074, which displayed a broad range of concentrations at rebound (Fig. 4g). These results are consistent with the idea that individuals who rebound do so when they develop resistance to 10-1074 and have subtherapeutic concentrations of 3BNC117 in circulation. Although the number of reservoir viruses analyzed was a small fraction of the total present in any given individual, the data indicate that the probability of escape from 10-1074 was directly related to the sensitivity of the intact proviruses in the initial reservoir (Fig. 4e). Furthermore, baseline reservoir size did not correlate with the presence or magnitude of autologous neutralizing activity against reservoir viruses, indicating that delayed rebound in bNAb-treated participants was not explained simply by a smaller baseline reservoir (Supplementary Fig. 1).

**Fig. 5:**
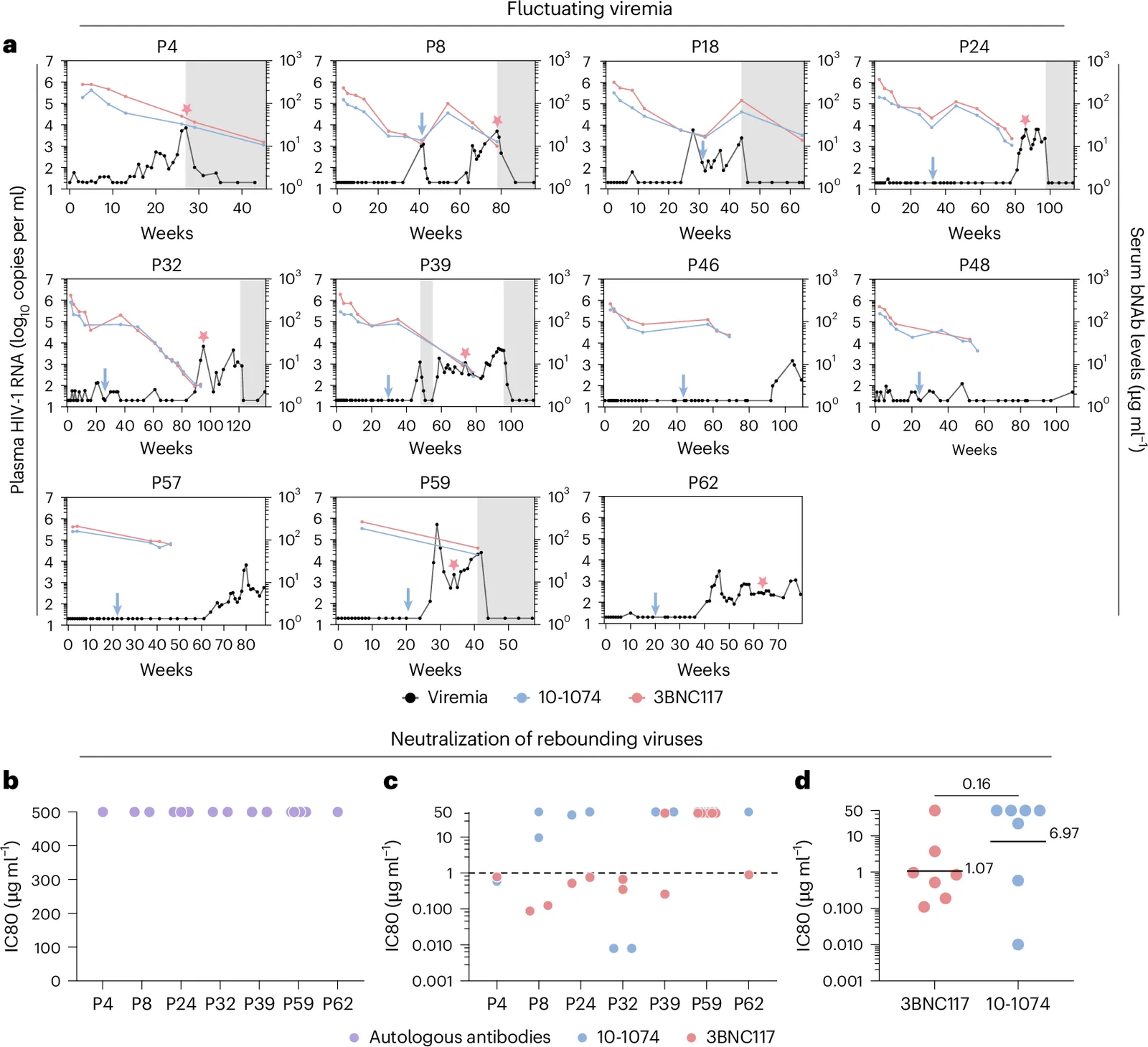
Virologic dynamics and antibody sensitivity during fluctuating viremia following bNAb therapy. **a**, Longitudinal plasma HIV-1 RNA levels (black) and circulating bNAb concentrations for 10-1074 (blue) and 3BNC117 (red) are shown for individual participants who maintained fluctuating viremia (defined as >16 weeks without sustained HIV-1 RNA >1,000 copies per ml). Blue arrows indicate administration of a second bNAb infusion. Gray shading denotes periods on ART. **b**, Neutralization sensitivity (IC80) of rebound-derived pseudoviruses to preinfusion autologous IgG for individual pseudoviruses. **c**, Neutralization sensitivity (IC80) of rebound-derived pseudoviruses to 3BNC117 (red) and 10-1074 (blue), shown for individual pseudoviruses. **d**, Each dot represents one participant (one biological replicate), using the geometric mean IC80 of all rebound-derived pseudoviruses from that participant against 3BNC117 or 10-1074 (*n* = 7 participants). Lines and numbers indicate geometric means across participants. Group comparisons were performed using a two-sided Wilcoxon matched-pairs signed-rank, with statistical significance defined as *P* < 0.05.

**Extended Data Fig. 8:**
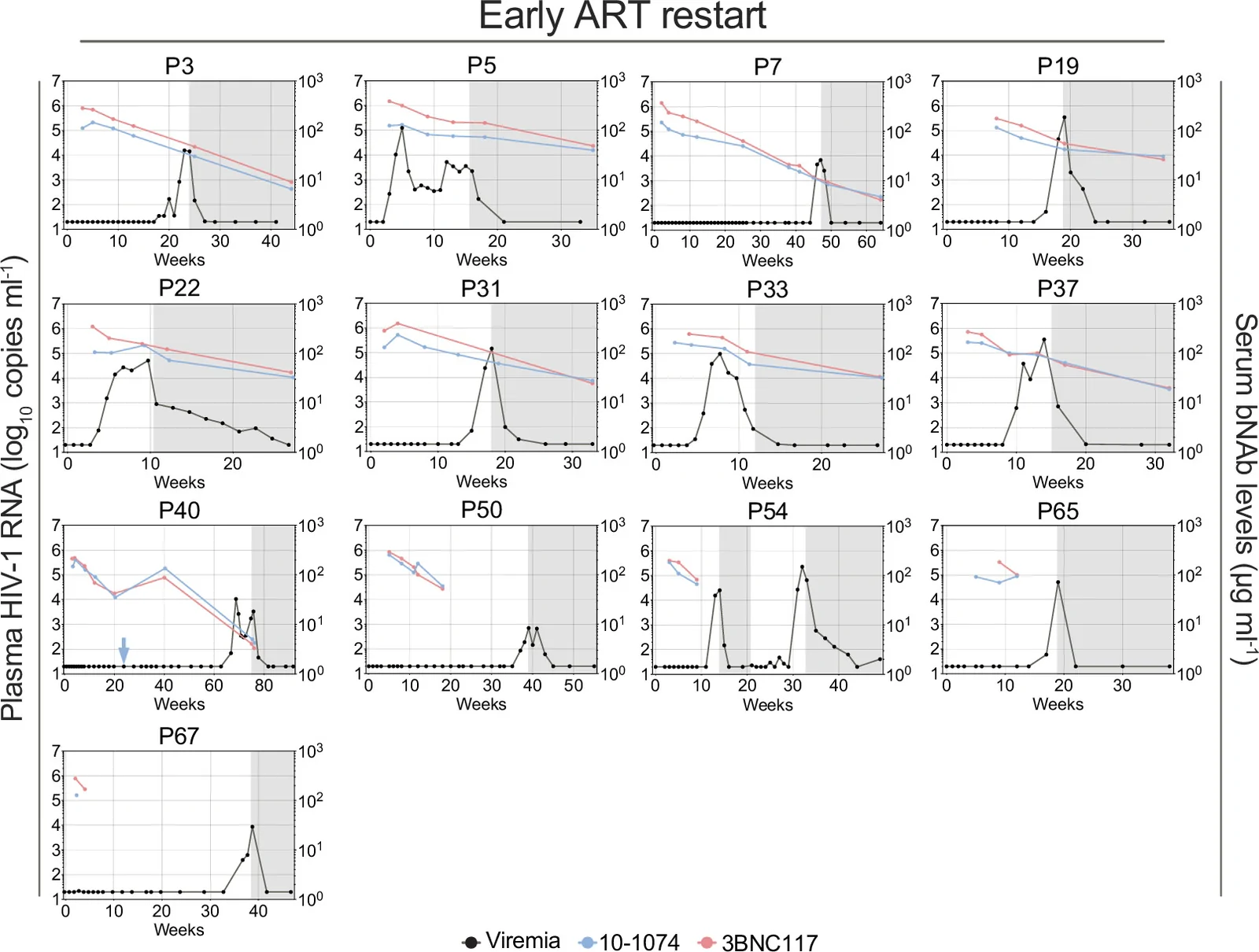
Longitudinal plasma viremia and circulating bNAb concentrations in participants with early ART restart. Longitudinal plasma HIV-1 RNA levels (black) and circulating bNAb concentrations for 10-1074 (blue) and 3BNC117 (red) are shown for participants who restarted ART early, defined by protocol criteria. Time is shown in weeks on the x axis, with HIV-1 RNA copies per milliliter and bNAb concentrations in microgram per milliliter plotted on log10 scales on the y axes. Blue arrows indicate administration of a second bNAb infusion. Gray shading denotes periods on antiretroviral therapy.

Among the 29 arm A participants who remained in follow-up according to protocol, 13 went back on ART before meeting virological restart criteria (two consecutive VLs ≥10^5^ HIV-1 copies per ml or six consecutive VLs ≥10^3^ HIV-1 copies per ml; Extended Data Fig. 8). In a post hoc subgroup analysis, of the 16 remaining, 11 (68%) maintained fluctuating viremia and did not meet ART restart criteria for 16 to >58 weeks (Fig. 5a). In contrast, only two of the arm B control showed prolonged fluctuating viremia^38^. We were able to obtain viruses from seven participants from time points associated with fluctuating viremia, and all were resistant to preinfusion autologous antibodies when present. In addition, five of these individuals showed viruses that were highly resistant to 10-1074 but retained sensitivity to 3BNC117 (Fig. 5c,d). These findings identify fluctuating viremia as a distinct post-bNAb virologic phenotype that others have recently associated with enhanced T cell immunity^24,29,31^.

### Reservoir dynamics

**Fig. 6:**
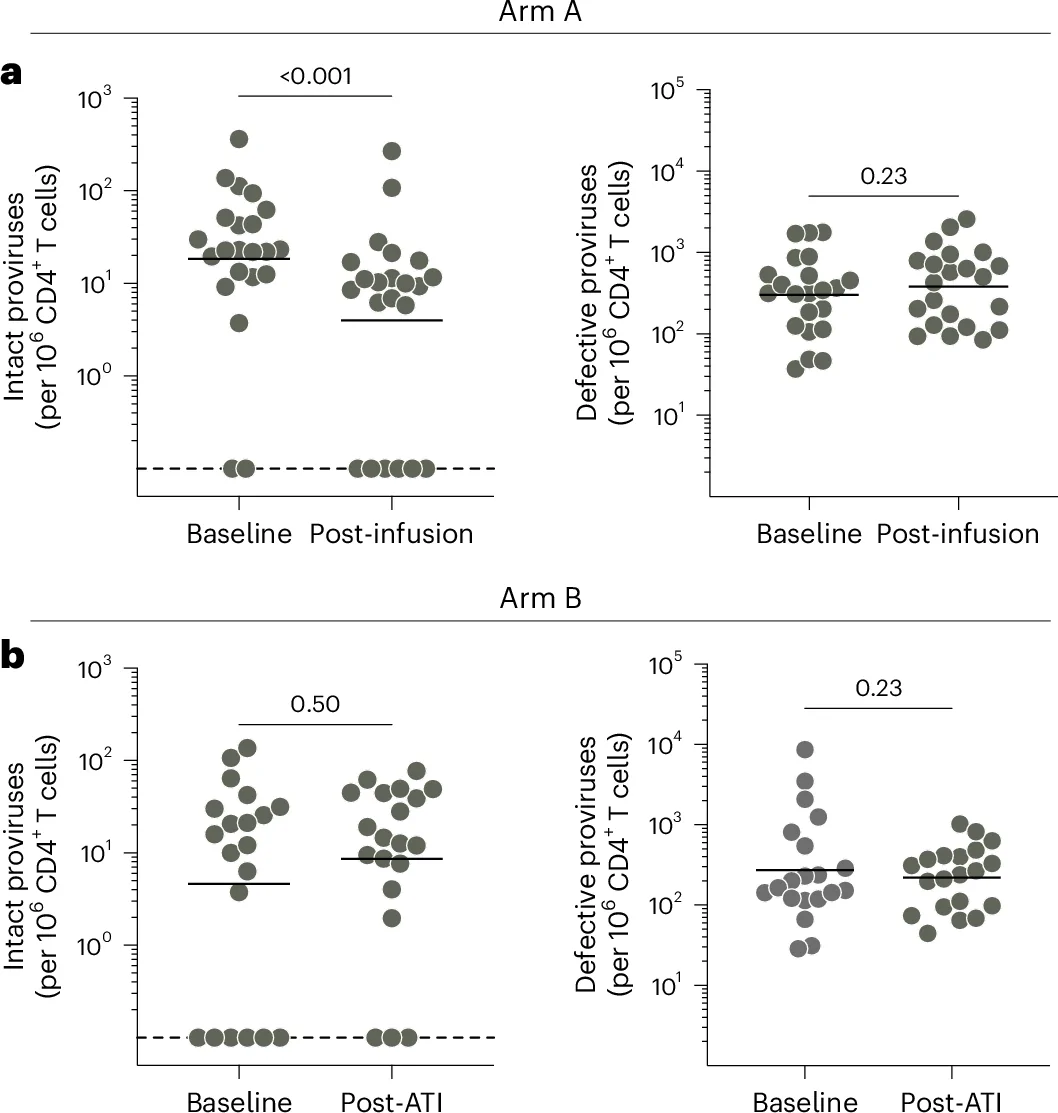
Intact and defective proviral reservoirs measured by ddPCR. **a**,**b**, Quantification of intact and defective proviral reservoirs by ddPCR at baseline and at follow-up time points in participants from arm A (*n* = 22) (**a**) and arm B (*n* = 20) (**b**). Each paired data point represents one participant (one biological replicate). Changes over time within each group were evaluated using a two-sided Wilcoxon matched-pairs signed-rank test (*P* < 0.05).

**Extended Data Fig. 9:**
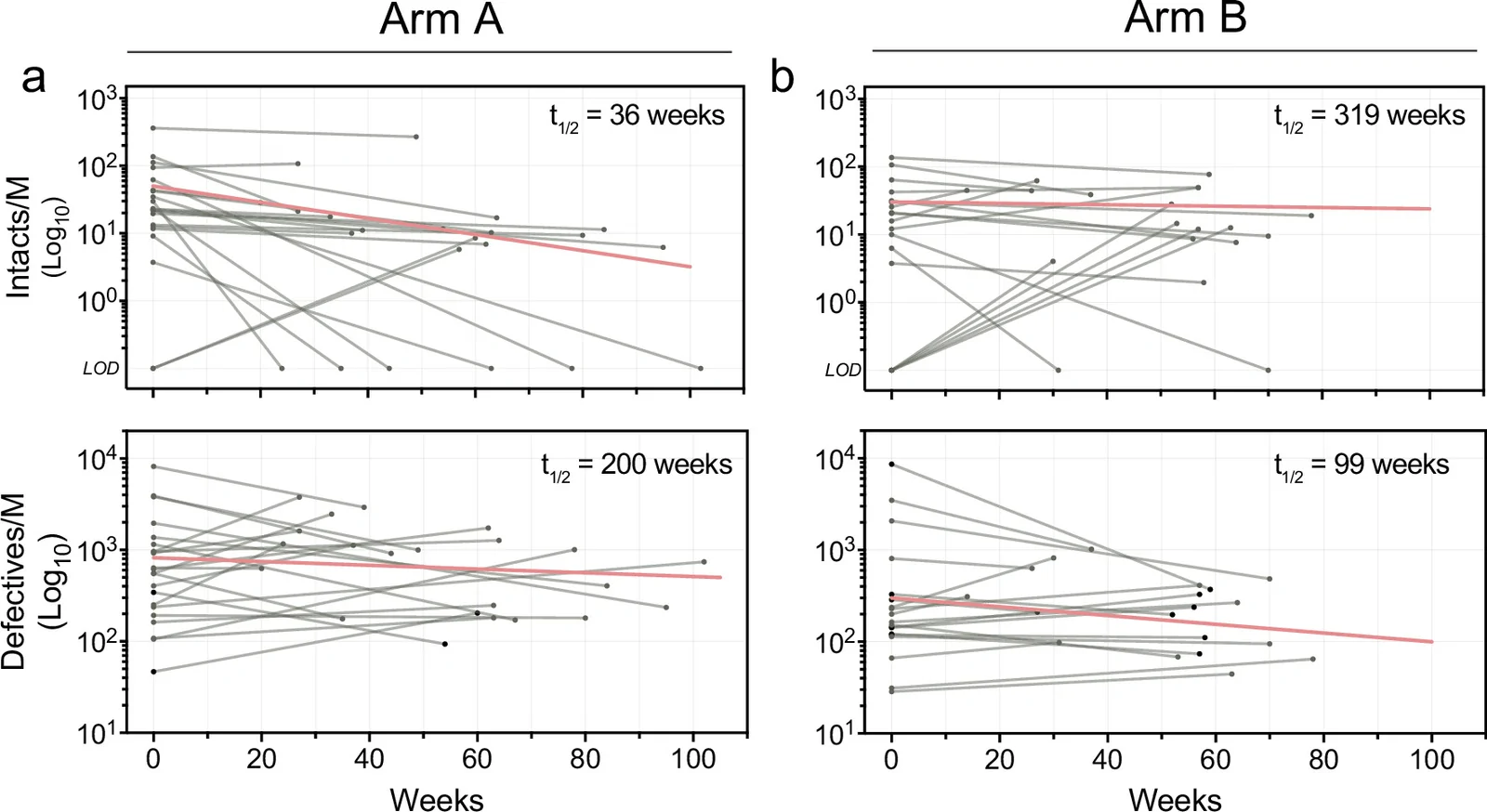
Decay of intact and defective proviral reservoirs measured by ddPCR. (**a,b**) Numbers of intact and defective proviruses quantified by ddPCR at baseline and follow-up time points in participants from Arm A (*n* = 22) and Arm B (*n* = 20). Time in weeks after the first infusion or treatment interruption is shown on the x axis. Each grey line represents one participant (one biological replicate). Half-life estimates were derived using log-linear regression of log_10_(*x* + 1)–transformed values; the red line indicates the fitted regression model.

To determine how the bNAbs impact the size of the proviral reservoir in arm A, we compared the intact and defective proviral reservoirs at baseline with prerebound samples collected at a mean of 52 weeks after the initial infusion (*n* = 22; Fig. 6 and Supplementary Table 1). Reservoir size was also measured in arm B after a mean of 52 weeks directly before the second treatment interruption^38^. We used ddPCR for these measurements because the relatively small reservoir size limited the number of samples from which we were able to obtain paired Q4PCR measurements (Supplementary Table 1). Although there was a great deal of variation between individuals in arm A over time, the intact reservoir but not the defective one was significantly smaller after bNAb therapy with an estimated half-life of 36 weeks (*n* = 22; Fig. 6a and Extended Data Fig. 9). In contrast, there was no measurable change in reservoir size in arm B (*n* = 20; Fig. 6b). As expected from the relatively small overall change in the intact reservoir in arm A, compared with estimated reservoirs of millions of cells, there was no correlation between the change in reservoir size and time to ART restart (Supplementary Fig. 2).

## Discussion

RIO is an ongoing double-blind, randomized placebo-controlled human trial in which participants who started ART during primary or early infection were randomly assigned to a group that received long-acting LS variants of 3BNC117 and 10-1074 (arm A) and a control group (arm B). Both groups subsequently discontinued ART^38^. Upon rebound, participants in arm B received ART and the two bNAbs before undergoing a second treatment interruption^38^. Despite similar preinfusion reservoir measurements, the groups differed significantly in time to rebound^38^ and in the nature of the rebounding viruses. After 96 weeks, 25% and 6% of the individuals in the active and control arms, respectively, remained off ART. Viral rebound occurred across a wide temporal range in arm A encompassing periods of high circulating bNAb concentrations as well as later time points when antibody levels were low. Because participants received the LS versions of the bNAbs, antibody exposure extended over long periods of time during ATI. Prolonged control was associated with pre-existing autologous neutralizing antibodies and sensitivity to 10-1074. Paired preinfusion and prerebound intact reservoir measurements showed a decrease in intact, but not defective, proviruses in arm A that was not observed in arm B participants. Notably, participants in arm B experienced viral rebound and periods of detectable viremia between sampling time points, which may have contributed to increase of the circulating reservoir. In addition, differences in baseline intact reservoir between the two arms may further confound these comparisons. Therefore, these associations are correlative and based on a limited number of individuals, but the observed reservoir decay is suggestive of altered reservoir dynamics in this cohort of PLWH undergoing ATI under LS-bNAb exposure.

As expected, based on their relatively early start on therapy, participants in RIO had small HIV-1 proviral reservoirs^41–43^. Nevertheless, most of the intact reservoir in participants was clonal and did not contribute to rebound^48,49^. Why reservoir proviruses are rarely found among rebound viruses is not entirely understood, but there is an inverse correlation between the size of an expanded clone and the probability that the sequence will be found among rebound viruses^12^. The difference has been attributed to selection for proviral integration into silent sites in the genome^18–20,53,54^, autologous antibody-mediated suppression^35,50^ and the possibility that rebound arises from tissue reservoirs not sampled in peripheral blood, as suggested by non-human primate studies^55,56^. Notably, the disparity between reservoir and rebound viruses was evident in control-arm individuals who did not have measurable autologous antibodies or receive bNAbs. Thus, the discrepancy between reservoir proviruses in circulation and rebound viruses cannot entirely be explained by autologous antibody suppression of viremia in this cohort of early treated individuals.

HIV-1 infection is associated with the development of antibodies that neutralize the autologous virus and continually select for viruses that express resistant variants during the course of the infection^33,34^. Moreover, autologous antibodies can sieve the outgrowth of reservoir viruses in vitro^35,50^. But although autologous antibodies alter the viral swarm by selection of resistant variants, they fail to control viremia, leading to the suggestion that antibodies are ineffective against HIV-1. This idea was further supported by the observation that passively administered first-generation bNAbs had little, if any, measurable effect on infection^57–59^. However, subsequent experiments in mice and macaques showed that administration of broader and more potent bNAbs suppresses viremia and elicits CD8^+^ T cell responses that lead to prolonged control of infection^21–23,60–62^. In addition, small interventional studies in which PLWH received second generation bNAbs demonstrated that antibodies can be both safe and effective in reducing viremia in the absence of ART^28,31,51,63–65^. Moreover, a fraction of individuals receiving bNAbs during treatment interruption appear to control the infection for an unexpectedly long time.

Enhanced CD8^+^ T cell responses were associated with bNAb therapy in some but not all reported clinical studies, and therefore other mechanisms are thought to contribute to enhanced control^24,30,31^. The RIO trial is a blinded placebo-controlled trial in which the data indicate that individuals treated early who have pre-existing autologous neutralizing antibodies are more likely to control for longer periods of time. The association between autologous antibodies and suppression is correlative and does not establish a direct mechanistic role for autologous antibodies in viral suppression. Persistent bNAbs may also contribute to prolonged control. Rather than acting independently, autologous antibodies may function as an adjunct to the infused antibodies, making viral escape from combined antibody pressure more difficult^66^. Notably, sensitivity to autologous antibodies correlated with sensitivity to 10-1074, suggesting partially overlapping aspects of viral neutralization susceptibility^67,68^. Several mechanisms by which antibodies can contribute to viral control have previously been documented, including direct neutralization of circulating viruses^69^, clearance of infected cells in vivo^61,70^ and antibody-mediated enhancement of antigen presentation to elicit cellular immune responses^71–73^. These mechanisms represent a plausible framework by which autologous antibodies could contribute to the delayed rebound observed in a subset of participants.

A substantial fraction of the participants in the bNAb arm of the RIO trial maintained fluctuating viremia for 16 to >58 weeks in the face of complete or partial bNAb resistance (11 of 29, or 38% of arm A). Although it did not reach significance, enhanced post-bNAb control has also been recently reported in the TITAN trial and in a single-arm study of ten individuals who received bNAbs, a Toll-like receptor agonist and a vaccine^24,29^. Similar results were also observed in bNAb-treated macaques^21–23^. Together, these observations point to fluctuating viremia as a reproducible post-bNAb virologic phenotype. The precise mechanistic explanation for these observations is not well defined, but the role of residual antibody as one contributing component cannot be dismissed.

Antibody monotherapy therapy like small-molecule therapy results in selection for resistant variants. Combination therapy durably suppresses viremia because the probability of developing escape mutations at a combination of non-overlapping different sites is lower. Consistent with this idea, rebound typically occurred in the RIO trial under functional monotherapy. Rebound viruses were most frequently resistant to 10-1074 and pre-existing autologous antibodies. In contrast, selection against 3BNC117 was far more limited, with no significant difference between initial reservoir and rebound sensitivity to the antibody, suggesting that the antibody was subtherapeutic at rebound. The median estimated 3BNC117-LS concentration at rebound was 52.4 μg ml^−1^, which is similar to the median 3BNC117 IC80 for these participants at the time of rebound (41.8 µg ml^−1^; Fig. 4f) and higher than the estimates in previous small clinical trials^25,27^. These findings further support the interpretation that fluctuating viremia and delayed rebound may not be explained by antibody effects alone.

RIO trial participants who received bNAbs demonstrated accelerated intact reservoir decay: 0.75 years compared with prior estimates of 4–7 years^14–17^. However, the accelerated intact reservoir decay was not uniform across the cohort, with 85% of the cohort showing a decrease. In contrast, there was no measurable change in reservoir in arm B between the first and second treatment interruption. The difference could be due to reservoir changes in response to viremia in arm B or the addition of ART to bNAb therapy in arm B or both. Accelerated reservoir decay was also a feature of a small study in which participants received multiple doses of a short-acting native version of 3BNC117 and 10-1074^25^, but not in other small studies^24,29^. The difference may be due to inherent variability and the small numbers of participants assayed, who were treated during chronic infection as opposed to early therapy participants in the RIO trial, differences in the antibodies used and their half-lives and finally substantial variability in the nature of the reservoir between individuals. Accordingly, we interpret intact reservoir decay in RIO as suggestive of altered reservoir dynamics under the specific conditions of early treatment and long-acting bNAb exposure. Why some participants in this trial showed only small or no change in reservoir size remains to be determined but may be due to heterogenous and/or variable levels of proviral transcription among CD4^+^ T cells and even among members of a clone of CD4^+^ T cells^74–76^. Moreover, the observed changes in reservoir size cannot account for and do not correlate with prolonged antibody-mediated suppression (Supplementary Fig. 2). Far greater changes in reservoir would be required to alter the time to rebound in most individuals.

In conclusion, the data establish that a substantial fraction of PLWH who receive bNAb therapy can partially control viremia for prolonged periods of time. Correlates of prolonged control of HIV-1 after bNAb therapy include pre-existing autologous antibodies and pre-existing or enhanced CD8^+^ T cell responses^29,36,52^, suggesting that vaccination to elicit or enhance pre-existing host immunity may further increase the number of individuals exhibiting post-bNAb control.

### Study limitations

Although 68 individuals were recruited into the RIO study, we were unable to obtain sequence information on the HIV-1 reservoir in 33 of the participants, thereby limiting the number of participant samples available for analysis. This constraint primarily reflects technical limitations of single-genome amplification in individuals treated early, who often harbor small circulating reservoirs, as well as issues related to sample availability and non-B clade viruses. Analyses of autologous neutralizing activity were restricted to participants with detectable reservoir-derived viral sequences. Moreover, sampling was limited to peripheral blood and may not entirely reflect tissue reservoirs. This problem was further accentuated when considering the relationship between autologous antibodies and virologic outcomes because early ART interferes with the development of autologous neutralizing antibody responses. Associations between autologous antibodies and delayed rebound are correlative, and reservoir decay estimates are limited by participants with values below assay detection. Finally, the timing of sample acquisition was predetermined and did not always correspond to time points of interest, limiting analysis of rebound viremia.

## Methods

### Ethics statement

This study complied with all relevant ethical regulations. The RIO trial was approved by the London–Westminster Research Ethics Committee (REC reference 19/LO/1669; IRAS ID 266322), the UK Health Research Authority and the UK Medicines and Healthcare products Regulatory Agency (MHRA reference 19174/0413/001) and the Danish Medicines Agency. The trial was registered under the European Union Drug Regulating Authorities Clinical Trials (EudraCT) database (2019-002129-31) and the European Union Clinical Trials Regulation (EU CT number 2024-514564-13-00) and registered at ClinicalTrials.gov (NCT04319367). The trial was conducted in accordance with the principles of the Declaration of Helsinki and Good Clinical Practice guidelines. All participants provided written informed consent before enrollment. Trial oversight included independent Data Monitoring and Trial Steering Committees that regularly reviewed participant safety and trial conduct.

### Study participants

The RIO trial is a randomized, placebo-controlled, double-blinded phase 2 study^37^. Participants were recruited across multiple HIV clinical research sites in the United Kingdom and one site in Denmark. Participants were randomly assigned in a 1:1 ratio using a centralized web-based randomization system with variable block sizes of two or four and no stratification. Participants, treating clinicians, investigators, study personnel and outcome assessors remained blinded to treatment allocation throughout the randomized phase of the trial.

This study enrolled individuals who initiated ART during confirmed primary HIV infection or during early HIV infection and remained on suppressive ART for at least 1 year. Primary infection was defined according to standard clinical and laboratory criteria at the time of diagnosis, as detailed in the RIO clinical protocol. Specifically, the estimated time of primary infection was determined by one of six criteria: (1) positive HIV-1 serology within 24 weeks of a documented negative HIV-1 serology result, with the estimated infection date defined as the midpoint between the negative and positive tests; (2) the date of a positive p24 antigen result, with or without a negative HIV antibody test, depending on local laboratory reporting; (3) the date of a negative HIV antibody test with detectable HIV RNA or proviral DNA; (4) a Public Health England Recent Infection Testing Algorithm result reported as ‘Incident,’ indicating antibody avidity consistent with infection within the preceding 16 weeks, with the estimated date of infection defined as 2 months before the incident test result, with the Asanté HIV-1 Rapid Recency Assay also permitted for recency testing; (5) the date of a weakly reactive or equivocal fourth-generation HIV antibody/antigen test; or (6) the date of an equivocal or reactive antibody test with fewer than four bands on western blot. Early-stage infection was operationally defined as ART initiation before substantial immune compromise, using a nadir CD4^+^ T cell count >500 cells per µl as a pragmatic surrogate for early disease stage.

Additional inclusion criteria included age 18–60 years, ability to provide informed consent including consent to long-term follow-up; willingness to comply with study visits and blood sampling; initiation of ART within 6 months of estimated primary HIV infection or during early HIV infection; virological suppression on ART for at least 1 year, with a single HIV-1 RNA measurement >50 but <500 copies per ml permitted during this period; no evidence of viral insensitivity to either 10-1074-LS or 3BNC117-LS; current CD4^+^ T cell count >500 cells per ml or CD4:CD8 ratio >1.0; nadir CD4^+^ T cell count >250 cells per µl for participants diagnosed with confirmed primary HIV infection; receipt of an integrase inhibitor (INSTI)- or boosted protease inhibitor (PI)-based ART regimen at randomization, with participants receiving a non-nucleoside reverse transcriptase inhibitor required to have switched to an INSTI- or boosted PI-based regimen at least 4 weeks before randomization; adequate hemoglobin (≥12 g dl^−1^ for males or ≥11 g dl^−1^ for females), body weight ≥50 kg; hepatitis B and C screening consistent with protocol eligibility (hepatitis B virus surface antigen or hepatitis B virus DNA negative and hepatitis C virus antigen or hepatitis C virus RNA negative or anticore antibody negative); absence of significant comorbidities; and SARS-CoV-2 vaccination at least 4 weeks before enrollment. Females capable of becoming pregnant were additionally required to use protocol-defined effective contraception or complete abstinence from at least 2 weeks before the first bNAb/placebo infusion until 20 months after the last bNAb infusion.

Exclusion criteria included significant cardiovascular disease, including previous ischemic heart disease (ST- or non-ST-elevation myocardial infarction, Q3-risk > 20, stable or unstable angina) or stroke; malignancy except squamous cell skin cancer; opportunistic infection or significant comorbidity or a comorbidity considered likely to occur during the trial, including malabsorption syndromes or autoimmune disease; contraindication to receipt of BHIVA-recommended combination ART; HTLV-1 co-infection; SARS-CoV-2 infection confirmed by RT–PCR up to 72 h before randomization/dosing where applicable according to local guidance; high risk of severe COVID-19 as determined according to applicable guidance and the participant’s physician; current or planned systemic immunosuppressive therapy (with inhaled and topical corticosteroids permitted); participation in another interventional trial of an experimental agent or a non-interventional study requiring additional blood draws, with observational studies permitted and non–clinical trials of investigational medicinal product studies involving minimal blood sampling considered on a case-by-case basis; history of severe reaction to antibody infusion or hypersensitivity to 3BNC117-LS, 10-1074-LS or their constituents or excipients; treatment with IV immunoglobulin or other monoclonal antibodies during the trial; clinically significant abnormal laboratory results including significant hepatic impairment with advanced fibrosis or cirrhosis with decompensation; alanine aminotransferase >5× the upper limit of normal; estimated glomerular filtration rate <60 ml per min per 1.73 m^2^; urine protein-to-creatinine ratio >30 mg mmol^−1^ or international normalized ratio >1.5; evidence of organ dysfunction or any clinically significant abnormality on physical examination or vital signs considered by the investigator to preclude enrollment; active alcohol or substance use likely to impair adherence; insufficient venous access; pregnancy or breastfeeding; or concern regarding risk-reduction precautions during ATI.

A total of 68 participants were enrolled between May 2021 and July 2024 as part of a two-stage clinical design. Clinical data and biospecimens used in this study were collected prospectively throughout trial follow-up between May 2021 and September 2025. Although the protocol permitted enrollment of both sexes, all participants enrolled in the trial were men, predominantly white. Participant disposition, including completion of follow-up, withdrawals and exclusions from specific analyses, is summarized in Extended Data Fig. 1. The RIO trial was powered for the primary clinical endpoint as described in the clinical paper^38^. Briefly, the study was designed to detect a reduction in the rebound rate at 36 weeks from 90% in arm B to 55% in arm A (hazard ratio 0.35), with 90% power at a 5% significance level. No formal power calculations were performed for the exploratory mechanistic analyses reported here. Sex and gender information were collected by self-report at study enrollment. Race and ethnicity information were self-reported according to local clinical site procedures. Because all enrolled participants were men, sex- and gender-based analyses were not performed. Participants received financial compensation for each visit. Blood samples were collected at baseline and at multiple time points following 3BNC117-LS and 10-1074-LS or placebo infusions. Samples were processed briefly after collection, with serum and plasma stored at −80 °C. PBMCs were isolated by density gradient centrifugation. The PBMC number was determined either manually or using an automated cell counter (Vi-Cell XR; Beckman Coulter), and cells were cryopreserved in liquid nitrogen in fetal bovine serum supplemented with 10% dimethyl sulfoxide.

### Definitions of virological control and ART-free status

Virological control was defined as sustained plasma HIV-1 RNA <1,000 copies per ml during treatment interruption. ART restart criteria were defined per protocol as either (1) two consecutive plasma HIV-1 RNA measurements > 10^5^ copies per ml or (2) six consecutive plasma HIV-1 RNA measurements > 10^3^ copies per ml. In addition to these virological criteria, ART could be restarted for other predefined clinical or participant-driven endpoints not meeting the above thresholds, including participant preference or clinician judgment. All ART restart events and their classification were adjudicated by an independent Endpoint Adjudication Committee. The distribution of ART restart outcomes by study arm is summarized in Extended Data Table 1.

### Intact proviral DNA analysis

The frequency of CD4^+^ T cells harboring intact, 5′-deleted and 3′-deleted proviruses was determined using a ddPCR assay modified from the Intact Proviral DNA Assay^10^. Briefly, CD4^+^ T cells were enriched from PBMCs using a negative immunomagnetic selection kit (Miltenyi Biotec). Genomic DNA was extracted using the QIAamp DNA Mini Kit (Qiagen) according to the manufacturer’s instructions. DNA concentrations were quantified using the Qubit 3.0 Fluorometer with the Qubit dsDNA BR Assay Kit (Thermo Fisher). Samples were from the preinfusion time point and obtained from an aviremic time from a minimum of 6 weeks before rebound, when HIV-1 RNA was <20 copies per ml.

For ddPCR, two sets of primers and probes specific to the HIV *gag* and *env* regions, along with two fragments of the housekeeping gene RPP30, were utilized in separate reactions^77^. HIV quantification was performed using 750 ng of genomic DNA per sample with the following primers and probes for the HIV *gag* region: forward (5′-GACTAGCGGAGGCTAGAAGGAGAGA-3′), reverse (5′-CTAATTCTCCCCCGCTTAATAYTGACG-3′) and probe (5′-6FAM-ATGGGTGCGAGA-IABkFQ-3′). For the HIV *env* region: forward (5′-AGTGGTGCAGAGAGAAAAAAGAGC-3′), reverse (5′-GTCTGGCCTGTACCGTCAGC-3′) and probes (5′-VIC-CCTTGGGTTCTTGGGA-MGB-3′ and an unlabeled hypermutated probe 5′-CCTTAGGTTCTTAGGAGC-MGB-3′). An alternative primer/probe set targeting the PS region was used as a backup: forward (5′-CAGGACTCGGCTTGCTGAAG-3′), reverse (5′-GCACCCATCTCTCTCCTTCTAGC-3′) and probe (5′-6FAM-TTTTGGCGTACTCACCAGT-IABkFQ-3′)^10^. To measure input cell numbers and correct for DNA shearing, 7.5 ng of DNA was used with RPP30 primers and probes (RPP30-1: forward (5′-GATTTGGACCTGCGAGCG-3′), reverse (5′-GCGGCTGTCTCCACAAGT-3′), probe (5′-6FAM-TTCTGACCTGAAGGCTCTGCGC-IABkFQ-3′); RPP30-2: forward (5′-GTGTGAGTCAATCACTAGACAGAA-3′), reverse (5′-AAACTGCAACAACATCATAGAGC-3′), probe (5′-HEX-AGAGAGCAACTTCTTCAAGGGCCC-IABkFQ-3′). Four technical replicates were performed per sample. Positive and negative controls were included in each reaction.

The ddPCR assays were conducted using the Bio-Rad QX200 AutoDG system with the ddPCR Supermix for Probes (no dUTPs) (Bio-Rad). Thermal cycling conditions included an initial denaturation at 95 °C for 10 min, followed by 45 cycles of 94 °C for 30 s and 59 °C for 1 min, with a 2 °C s^−1^ ramp rate. A final extension was performed at 98 °C for 10 min, followed by a hold at 12 °C. The results were adjusted for DNA shearing using the ratio of double-positive RPP30 partitions and normalized to 10^6^ CD4^+^ T cells.

### Q4PCR

The Q4PCR assay was conducted to characterize the composition of the reservoir, as previously described^11^. Briefly, total CD4^+^ T cells were enriched from cryopreserved PBMCs using a negative immunomagnetic selection kit (Miltenyi Biotec). Genomic DNA was extracted from CD4^+^ T cells using the Gentra Puregene Cell Kit (Qiagen), and DNA concentration was measured using the Qubit dsDNA BR Assay Kit (Thermo Fisher).

A total range of 1 to 10 × 10^6^ CD4 T cells were screened per participant. An initial outer PCR (near full length, step 1 (NFL1)) was performed on genomic DNA at a single-copy dilution using the outer primers BLOuterF (5′-AAATCTCTAGCAGTGGCGCCCGAACAG-3′) and BLOuterR (5′-TGAGGGATCTCTAGTTACCAGAGTC-3′)^78^. A 1-µl aliquot of the undiluted NFL1 PCR product was then subjected to a Q4PCR reaction, utilizing four primer–probe sets targeting conserved regions of the HIV-1 genome: PS: forward (5′-TCTCTCGACGCAGGACTC-3′), reverse (5′-TCTAGCCTCCGCTAGTCAAA-3′), probe (5′-/Cy5/TTTGGCGTA/TAO/CTCACCAGTCGCC-3′/IAbRQSp); *env*: forward (5′-AGTGGTGCAGAGAGAAAAAAGAGC-3′), reverse (5′-GTCTGGCCTGTACCGTCAGC-3′), probe (5′-/VIC/CCTTGGGTTCTTGGGA-3′/MGB); *gag*: forward (5′-ATGTTTTCAGCATTATCAGAAGGA-3′), reverse (5′- TGCTTGATGTCCCCCCACT-3′), probe (5′-/6-FAM/CCACCCCAC/ZEN/AAGATTTAAACACCATGCTAA-3′/IABkFQ); and pol: forward (5′-GCACTTTAAATTTTCCCATTAGTCCTA-3′), reverse (5′-CAAATTTCTACTAATGCTTTTATTTTTTC-3′), probe (5′-/NED/AAGCCAGGAATGGATGGCC-3′/MGB). Quantitative PCR was performed under the following thermal cycling conditions: an initial denaturation at 94 °C for 10 min, followed by 40 cycles of 94 °C for 15 s and 60 °C for 60 s. Quantitative PCR assays were conducted in a 384-well plate format using the Applied Biosystem QuantStudio 6 or 7 Flex real-time PCR system. Data analysis was performed using Thermo Fisher Design and Analysis Software 2.4.3. Samples that exhibited reactivity with two or more of the four probes were selected for a nested PCR (NFL2).

The NFL2 reaction was performed on undiluted 1-µl aliquots of the NFL1 PCR product. Each reaction was conducted in a 20-µl volume using Platinum Taq high-fidelity polymerase (Thermo Fisher) and the primers 3LTRi (5′- TCAAGGCAAGCTTTATTGAGGCTTAA-3′) and U5-638F (5′- GCGCCCGAACAGGGACYTGAAARCGAAAG-3′)^19^. The thermocycler conditions for NFL2 were the same as those used for the NFL1 PCR. Library preparation and sequencing were conducted as previously described^11^.

### HIV-1 sequence assembly and annotation

HIV-1 genome reconstruction was performed using an in-house pipeline, Defective and Intact HIV Genome Assembler, designed for assembling raw sequencing reads into annotated HIV genomes^25^. The pipeline includes rigorous quality control steps to enhance accuracy and reliability. First, quality control checks were conducted to remove PCR-amplified reads and correct sequencing errors using clumpify.sh from the BBtools package v38.72 (http://sourceforge.net/projects/bbmap). Next, adapter sequences and low-quality bases were trimmed, and potential contaminant reads were removed using the Trim Galore package v0.6.4 (https://github.com/FelixKrueger/TrimGalore). HIV-1 sequences were assembled using SPAdes v3.13.0, and the longest assembled contig was aligned to the HXB2 HIV-1 reference genome via BLAST^79^. Sequences exhibiting double peaks—regions indicating the presence of two or more viral variants within a sample (defined by a consensus identity cut-off of <70% for any residue)—or samples with an insufficient number of sequencing reads (≤500 reads) were excluded from downstream analyses.

Sequences that did not meet the double-peak criteria (consensus identity for any residue <70%) were further classified as either intact or defective proviruses. Only intact HIV-1 *env* sequences were considered for subsequent analyses.

### Single-genome amplification of plasma rebound virus *env* genes

Sequencing of HIV-1 plasma rebound *env* genes was performed as previously described^80^. In brief, HIV-1 RNA was extracted from viremic plasma samples using the MinElute Virus Spin kit (Qiagen) according to the manufacture’s recommendation. First-strand cDNA synthesis was carried out using SuperScript III reverse transcriptase (Invitrogen) and an antisense primer, envB3out (5′-TTGCTACTTGTGATTGCTCCATGT-3′) for subtype B or OFM19 (5′-GCACTCAAGGCAAGCTTTATTGAGGCTTA-3′) for subtype C. To ensure single-genome amplification, cDNA was endpoint diluted according to Poisson distribution, achieving <30% of wells yielding a PCR product. The envelope gene was then PCR-amplified using subtype B primers envB3out and envB5out (5′-TAGGCATCTCCTATGGCAGGAAGAAG-3′) or subtype C primers OFM19 and Vif1 (5′-GGTTTATTACAGGGACAGCAGAG-3′). A second round of PCR was performed using 1 µl of the first-round PCR product as a template, with subtype B primers envB3in (5′-GTCTCGAGATACTGCTCCCACCC-3′) and envB5in (5′-TAGGCATCTCCTATGGCAGGAAGAAG-3′) or subtype C primers *env* A (5′-GGCTTAGGCATCTCCTATGGCAGGAAGAA-3′) and *env* N (5′-CTGCCAATCAGGGAAGTAGCCTTGTGT-3′). PCR amplifications were conducted using High Fidelity Platinum Taq (Invitrogen) under the following conditions: an initial denaturation at 94 °C for 2 min, followed by 35 cycles of 94 °C for 15 s, 55 °C for 30 s and 68 °C for 4 min, with a final extension at 68 °C for 10 min. PCR products of the expected size were subjected to library preparation and sequencing using the Illumina MiSeq platform.

### Sequence and phylogenetic analysis

Nucleotide alignments of intact *env* sequences were performed using Muscle v5.1 with the PPP algorithm^81^. Sequences containing premature stop codons, truncations or frameshift mutations were excluded from further analyses. Maximum-likelihood phylogenetic trees were generated from these alignments using FastTree 2.1.11 with the GTR model and 1,000 bootstrap replicates^82^.

### Pseudotyped-virus production

Selected single-genome sequences from CD4^+^ T cell reservoir or plasma rebound samples were used as templates to produce pseudoviruses from a cytomegalovirus (CMV) promoter as described^83^. The CMV promoter was amplified by PCR from pcDNA 3.1 (Life Technologies) using the primers CMVenv (5′-AGTAATCAATTACGGGGTCATTAGTTCAT-3′) and CMVenv1A (5′-CATAGGAGATGCCTAAGCCGGTGGAGCTCTGCTTATATAGACCTC-3′). Thermocycling conditions were 94 °C for 2 min, followed by 30 cycles of 94 °C for 30 s, 55 °C for 30 s and 68 °C for 4 min. A 1-µl aliquot of the second-round PCR product from NFL amplification (reservoir) or single-genome amplification (rebound) was used as a template for *env* to which we added CMV overhanging regions using the forward primer ENVfwd (5′-CACCGGCTTAGGCATCTCCTATGGCAGGAAGAA-3′) and the reverse primer envB3in or ENV N, depending on the subtype. The CMV promoter amplicon was fused to individual *env* genes via overlapping PCR with 10 ng of *env* and 0.5 ng of CMV using CMVenv primer and envB3in or ENV N as the reverse primer. Thermocycling conditions were 94 °C for 2 min followed by 20 cycles of 94 °C for 30 s, 55 °C for 30 s, 68 °C for 4 min, followed by a final extension at 68 °C for 10 min. All PCR reactions were carried out using Platinum Taq HiFi polymerase. Resulting amplicons were analyzed by gel electrophoresis, purified using the Macherey-Nagel gel and PCR purification kit and cotransfected with the pSG3Δ*env* vector (National Institutes of Health AIDS Reagent Program) into 293T cells to produce pseudoviruses as previously described.

### Neutralization assays

Viruses were tested against bNAbs and purified autologous IgGs using the TZM-bl cell neutralization assay with cells obtained through the National Institutes of Health HIV Reagent Program, as previously described^47,84^. Briefly, pseudoviruses were incubated with eight fivefold serial dilutions of antibodies for 1 h at 37 °C in 96-well plates before addition of 10,000 TZM-bl cells per well in growth medium containing DEAE-dextran. Cells were incubated for 48 h at 37 °C in 5% CO_2_, after which infection was quantified by measuring luciferase activity using Bright-Glo reagent (Promega). Neutralization was calculated relative to virus-only control wells, and IC values were determined by nonlinear regression. Assays were performed in duplicate under Good Clinical Laboratory Practice quality assurance standards. For bNAb testing, the starting antibody concentrations were 50 μg ml^−^^1^ for 3BNC117 and 10-1074, and purified autologous IgG was tested at an initial concentration of 500 μg ml^−^^1^ followed by eight fivefold serial dilutions. Pseudovirus clones derived from both the reservoir and rebound were tested. For autologous antibody testing, baseline IgG was purified using Protein G Sepharose 4 Fast Flow (GE Life Sciences). Neutralization breadth was evaluated using a panel of ten HIV-1 *env* pseudoviruses selected based on reported resistance to both 3BNC117 and 10-1074 (https://www.hiv.lanl.gov/catnap). The panel included the following *env* clones: 700010040.C9.4520, H030.7, H086.8, THRO4156.18, H035.18, H704_1109_140_RE_cs, 191955_A11, 89-F1_2_25, 6322.V4.C1 and 3637.V5.C3.

### Depletion of residual bNAbs from longitudinal IgG samples

To remove residual bNAbs from longitudinal IgG samples, anti-3BNC117 and anti-10-1074 antibodies engineered with a FLAG epitope on the light chain were used as capture reagents. Following IgG purification from plasma, samples were incubated with the FLAG-tagged anti-bNAb-idiotype reagents at a twofold molar ratio of the anti-idiotype to the estimated bNAb concentration for 2 h at 4 °C to allow immune complex formation. Samples were then incubated overnight at 4 °C with anti-FLAG M2 magnetic beads (Sigma-Aldrich, catalogue number M8823). Beads were removed magnetically, and the supernatant (depleted of infused bNAbs) was collected, tested for residual bNAbs by ELISA and, if below the limit of detection, used for downstream autologous neutralization assays. Depletion was repeated if residual bNAb was detected until it no longer was.

### Time-to-event analyses

To evaluate the combined influence of endogenous and therapeutic antibody pressure on time to ART restart, time-to-event analyses were performed using Cox proportional hazards models with time to ART restart as the outcome. Cox proportional hazards analyses incorporated observed ART restart events occurring beyond 96 weeks when available. Baseline reservoir sensitivity to autologous antibodies, 10-1074 and 3BNC117 was quantified using IC80 values, which were log_10_-transformed and standardized before modeling. Univariable, pairwise (models including two resistance variables simultaneously) and multivariable models were fit to assess the individual and combined contributions of each resistance dimension to rebound timing. Hazard ratios reflect the effect of a one-standard-deviation increase in resistance. Analyses were performed using Cox proportional hazards regression in GraphPad Prism and R (survival package version 3.8-6).

### Statistics and reproducibility

Statistical analyses were performed using GraphPad Prism version 10.6.1 (GraphPad Software) and R version 4.5.0 (R Foundation for Statistical Computing), as described for each analysis. All statistical tests were two-sided unless otherwise specified. Exact statistical tests, sample sizes, measures of central tendency and definitions of biological replicates are provided in the corresponding figure legends. No statistical method was used to predetermine sample size for laboratory analysis because sample size was determined by participant enrollment and sample availability within the RIO clinical trial. No data were excluded from the analyses unless explicitly stated. Statistical analyses of prespecified secondary and exploratory outcomes were conducted according to the trial Statistical Analysis Plan. Analyses not prespecified in the Statistical Analysis Plan are explicitly identified in ‘Results’ as non-prespecified exploratory or post hoc analyses. Participants were randomized as part of the RIO clinical trial according to the study protocol. Laboratory investigators performing the mechanistic analyses were initially blinded to treatment allocation. Blinding was maintained until participants met protocol-defined criteria for unblinding (for example, following viral rebound), after which treatment allocation became known for subsequent analyses. Each participant was considered one biological replicate unless otherwise indicated, and technical replicates were not treated as independent observations. Correlation analyses were performed using two-sided Spearman rank correlation coefficients with 95% confidence intervals. Group comparisons were performed using two-sided Mann–Whitney tests or Wilcoxon matched-pairs signed-rank tests, as appropriate. Time-to-event analyses were evaluated using Cox proportional hazards regression. Statistical significance was assessed using exact two-sided *P* values, which are reported in the figures or text.

### Reporting summary

Further information on research design is available in the Nature Portfolio Reporting Summary linked to this article.

## Online content

Any methods, additional references, Nature Portfolio reporting summaries, source data, extended data, supplementary information, acknowledgements, peer review information; details of author contributions and competing interests; and statements of data and code availability are available at https://doi.org/10.1038/s41591-026-04644-8.

## Supplementary information

Supplementary Information Supplementary Figs. 1 and 2.

Reporting Summary

Peer Review File

**Supplementary Table 1** Quantification of HIV reservoir size by ddPCR and quantitative Q4PCR for individual participants, **Supplementary Table 2** Neutralization sensitivities and baseline near full-length proviral sequences from participants in arms A and B, **Supplementary Table 3** Neutralization sensitivities and individual *env* coding region sequences obtained from plasma of participants in arms A and B during rebounding viremia, **Supplementary Table 4** Geometric mean neutralization sensitivities of reservoir-derived and rebound pseudoviruses from individual participants in both study arms, tested against baseline autologous IgG, 3BNC117 and 10-1074, **Supplementary Table 5** Serum concentrations of 3BNC117-LS and 10-1074-LS in participants with prolonged viral control, **Supplementary Table 6** Cox proportional hazards models evaluating the relationship between time to viral rebound and baseline reservoir sensitivity to autologous antibodies, 10-1074 and 3BNC117

## Data Availability

Source data underlying the figures and statistical analyses presented in this study are provided in Supplementary Tables. Specifically, participant-level reservoir quantification data are provided in Supplementary Table 1, baseline intact reservoir-derived proviral sequences and neutralization data in Supplementary Table 2, rebound virus *env* sequences and neutralization data in Supplementary Table 3, participant-level geometric mean neutralization sensitivities in Supplementary Table 4, serum concentrations of 3BNC117-LS and 10-1074-LS of long-term controllers in Supplementary Table 5, and Cox proportional hazards model outputs in Supplementary Table 6. HIV-1 sequence data generated for all reservoir-derived proviral genomes in this study have been deposited in GenBank under accession numbers PZ799384–PZ799601 and PZ799607–PZ802566. Newly generated research materials, including participant-derived HIV-1 *env* constructs, pseudovirus reagents and other unique materials generated in this study, will be made available to qualified researchers upon reasonable request to the corresponding author, subject to applicable institutional approvals, material transfer agreements, participant consent restrictions and the continued availability of the requested materials. Deidentified participant-level clinical and laboratory data from the RIO trial, including relevant data dictionaries and supporting study documentation, will be available through a controlled-access process following completion of the trial and database lock, in accordance with participant consent and institutional ethical requirements. Clinical data requests for access should be directed to S. Fidler (s.fidler@imperial.ac.uk), University of Oxford, who serves as chief investigator of the RIO trial. Requests will be acknowledged within ten working days and reviewed by the Trial Management Group, Independent Data Monitoring Committee and Trial Steering Committee. A decision regarding data access will normally be communicated within 8 weeks of receipt of a complete request. Approved investigators will be required to sign a data access agreement before receiving access to the requested datasets. The RIO clinical protocol and Statistical Analysis Plan are available as supplementary documents accompanying the primary clinical publication^38^.

## Extended data

**Extended Data Table 1:** Baseline demographic and clinical characteristics of RIO participants

Characteristic	Arm A: bNAbs (n = 34)	Arm B: Placebo (n = 34)	All (n = 68)
Age, years (Median, IQR)	36 (31, 44)	42 (33, 51)	40 (32, 46)
Male sex assigned at birth, n (%)	34 (100.0%)	34 (100.0%)	68 (100.0%)
Asian or Asian British, n (%)	3 (8.8%)	0 (0.0%)	3 (4.4%)
Black or Black British, n (%)	1 (2.9%)	2 (5.9%)	3 (4.4%)
White ethnicity, n (%)	28 (82.4%)	30 (88.2%)	58 (85.3%)
Other ethnicity, n (%)	2 (5.9%)	2 (5.9%)	4 (5.9%)
Baseline CD4 count, cells per µl (Median, IQR)	742 (657, 937)	810 (596, 986)	776 (626, 963)
HIV clade B, n (%)	19 (76.0%)	21 (84.0%)	40 (80.0%)
Non-B clade, n (%)	6 (24.0%)	4 (16.0%)	10 (20.0%)
Clade information unavailable, n	9	9	18
Duration on ART before enrolment, years (Median, IQR)	5 (3–6)	5 (4–7)	5 (4–7)
Primary HIV infection, n (%)	32 (94%)	30 (88%)	62 (91%)
Early HIV infection, n (%)	2 (6%)	4 (12%)	6 (9%)
