# Supplementary material for: Broadly neutralizing antibodies in adult males living with HIV undergoing analytical treatment interruption: secondary and exploratory outcomes of the phase II randomized controlled RIO trial
Source: Nat Med. 2026 Sep 15;32(11):3990–4001. doi: 10.1038/s41591-026-04644-8 (PMC13768170; doi:10.1038/s41591-026-04644-8)
Supplement: Supplementary file 1 [file 41591_2026_4644_MOESM1_ESM.pdf]

nature medicine

Supplementary information

https://doi.org/10.1038/s41591-026-04644-8

Broadly neutralizing antibodies in adult males living with HIV undergoing analytical treatment interruption: secondary and exploratory outcomes of the phase II randomized controlled RIO trial

In the format provided by the authors and unedited

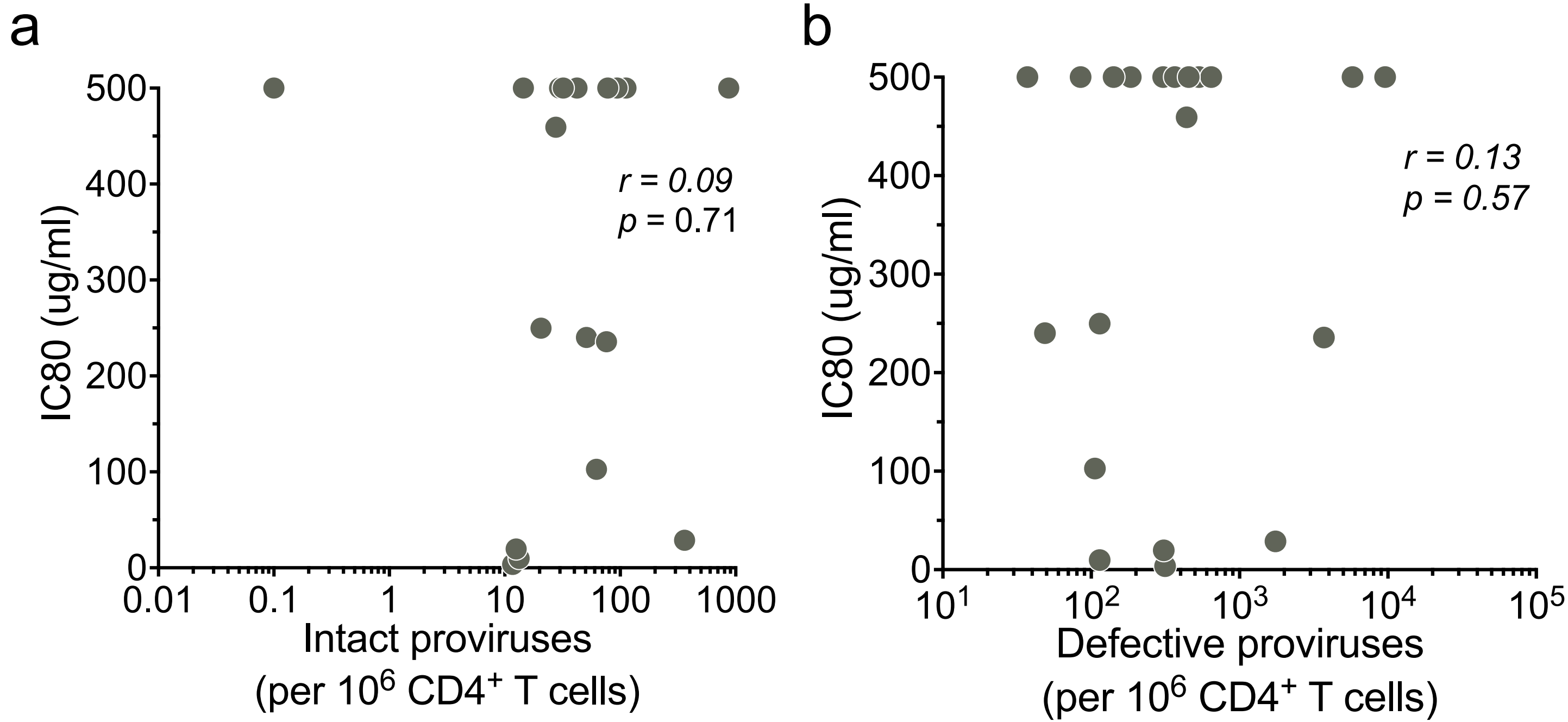

Supplementary Fig. 1. Relationship between baseline reservoir size and autologous neutralizing activity. Baseline (a) intact and (b) defective proviral DNA levels measured by ddPCR are shown for participants with and without detectable autologous neutralization against reservoir-derived viruses. Correlation coefficients were calculated using Spearman's r with 95% confidence intervals.

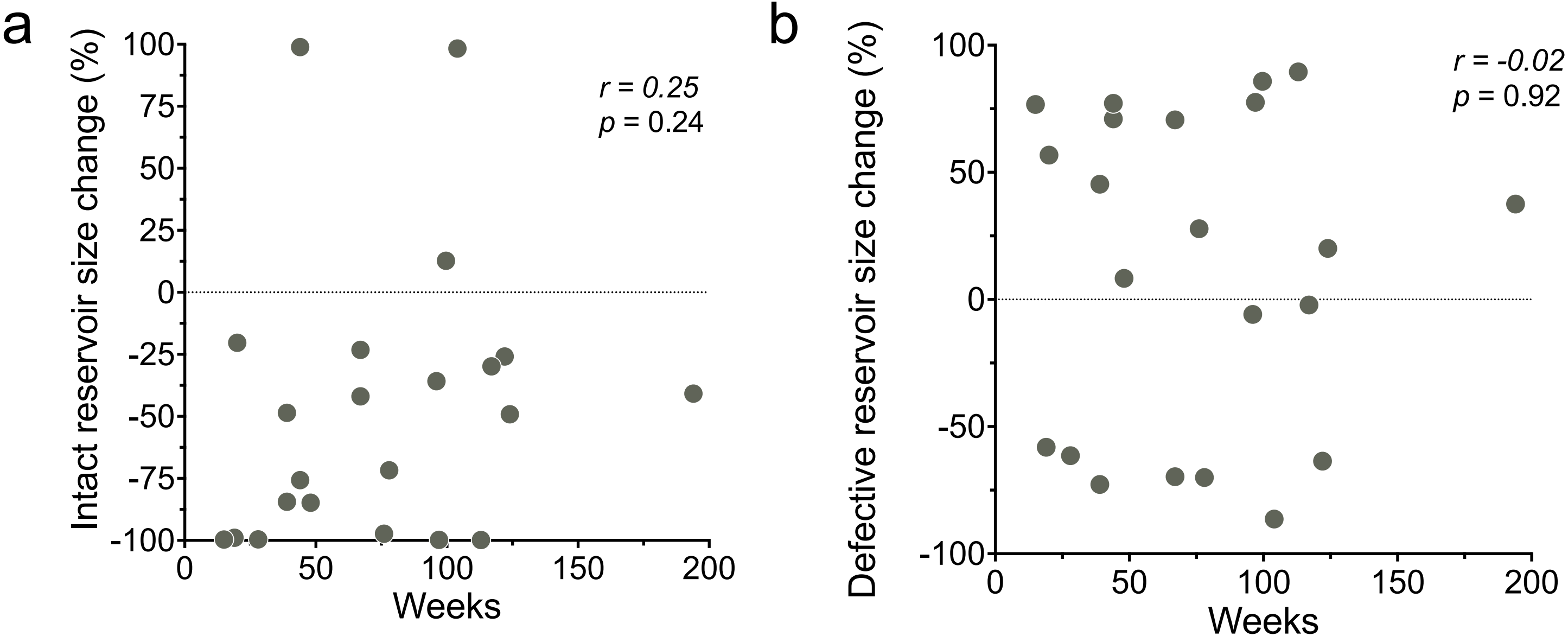

Supplementary Fig. 2. Correlation between the change in reservoir size and time to ART restart. Scatter plots showing the relationship between time to ART restart and the change in (a) intact and (b) defective reservoir size relative to the pre-infusion baseline. The x axis indicates time to ART (weeks) and the y axis indicates the percentage change in reservoir size. Each point represents an individual participant. The dotted line indicates 0% change relative to baseline. Correlation coefficients were calculated using Spearman's r with 95% confidence intervals
