# Supplementary material for: Broadly neutralizing antibodies in adult males living with HIV undergoing analytical treatment interruption: secondary and exploratory outcomes of the phase II randomized controlled RIO trial
Source: Nat Med. 2026 Sep 15;32(11):3990–4001. doi: 10.1038/s41591-026-04644-8 (PMC13768170; doi:10.1038/s41591-026-04644-8)
Supplement: Supplementary file 2 [file 41591_2026_4644_MOESM2_ESM.pdf]

nature portfolio

Corresponding author(s): Michel C. Nussenzweig

Last updated by author(s): Aug 9, 2026

Reporting Summary

Nature Portfolio wishes to improve the reproducibility of the work that we publish. This form provides structure for consistency and transparency in reporting. For further information on Nature Portfolio policies, see our Editorial Policies and the Editorial Policy Checklist.

Statistics

For all statistical analyses, confirm that the following items are present in the figure legend, table legend, main text, or Methods section.

n/a	Confirmed	
☐	☒	The exact sample size (*n*) for each experimental group/condition, given as a discrete number and unit of measurement
☐	☒	A statement on whether measurements were taken from distinct samples or whether the same sample was measured repeatedly
☐	☒	The statistical test(s) used AND whether they are one- or two-sided<br>*Only common tests should be described solely by name; describe more complex techniques in the Methods section.*
☒	☐	A description of all covariates tested
☐	☒	A description of any assumptions or corrections, such as tests of normality and adjustment for multiple comparisons
☐	☒	A full description of the statistical parameters including central tendency (e.g. means) or other basic estimates (e.g. regression coefficient) AND variation (e.g. standard deviation) or associated estimates of uncertainty (e.g. confidence intervals)
☐	☒	For null hypothesis testing, the test statistic (e.g. F, t, r) with confidence intervals, effect sizes, degrees of freedom and P value noted<br>*Give P values as exact values whenever suitable.*
☒	☐	For Bayesian analysis, information on the choice of priors and Markov chain Monte Carlo settings
☒	☐	For hierarchical and complex designs, identification of the appropriate level for tests and full reporting of outcomes
☐	☒	Estimates of effect sizes (e.g. Cohen's *d*, Pearson's *r*), indicating how they were calculated

Our web collection on statistics for biologists contains articles on many of the points above.

Software and code

Policy information about availability of computer code

Data collection	Instrumentation & Associated Acquisition Software<br>1. Applied Biosystems QuantStudio 6 / 7 Flex Real-Time PCR System - (qPCR data acquisition)<br>2. Bio-Rad QX200 AutoDG System - (ddPCR droplet generation + data acquisition)<br>3. Illumina MiSeq Sequencing Platform - (next-generation sequencing data generation)<br>4. Beckman Coulter Vi-Cell XR Automated Cell Counter - (cell number acquisition)<br>5. FLUOstart Omega microplate reader - (Luminescence reading)
Data analysis	Commercial software<br>Thermo Fisher Design and Analysis Software v2.4.3 (Thermo Fisher Scientific) – qPCR/ddPCR data analysis.<br>GraphPad Prism v10.6.1 (GraphPad Software, LLC) – statistical analyses and graph generation.<br>Adobe Illustrator (Adobe Inc.) – figure assembly and preparation.<br>Interactive Tree of Life (iTOL) v6 – phylogenetic tree visualization and annotation.<br>Open-source and bioinformatics software<br>MUSCLE v5.1 – multiple sequence alignment.<br>FastTree v2.1.11 – maximum-likelihood phylogenetic tree reconstruction.<br>BBTools (clumpify.sh) v38.72 – read deduplication and error correction.<br>Trim Galore v0.6.4 – adapter trimming and quality filtering.<br>SPAdes v3.13.0 – genome assembly.<br>BLAST+ (NCBI) – sequence alignment and similarity searches.<br>R v4.5.0 (R Foundation for Statistical Computing) – statistical analyses.

Custom software
DIHIVA pipeline (Defective and Intact HIV Genome Assembler) – custom pipeline developed for HIV proviral genome assembly and annotation.

For manuscripts utilizing custom algorithms or software that are central to the research but not yet described in published literature, software must be made available to editors and reviewers. We strongly encourage code deposition in a community repository (e.g. GitHub). See the Nature Portfolio guidelines for submitting code & software for further information.

Data

Policy information about availability of data

All manuscripts must include a data availability statement. This statement should provide the following information, where applicable:

- Accession codes, unique identifiers, or web links for publicly available datasets
- A description of any restrictions on data availability
- For clinical datasets or third party data, please ensure that the statement adheres to our policy

Source data underlying all figures and statistical analyses are provided in the Supplementary Tables accompanying this manuscript. Participant-level reservoir quantification data are provided in Supplementary Table 1; baseline intact reservoir-derived proviral sequences and neutralization data in Supplementary Table 2; rebound virus env sequences and neutralization data in Supplementary Table 3; participant-level geometric mean neutralization sensitivities in Supplementary Table 4; serum concentrations of 3BNC117-LS and 10-1074-LS in long-term controllers in Supplementary Table 5; and Cox proportional hazards model outputs in Supplementary Table 6. HIV-1 sequence data generated for all reservoir-derived proviral genomes have been deposited in GenBank.

Because the study involves human participants enrolled in the RIO clinical trial, de-identified participant-level clinical and laboratory datasets are available through a controlled-access process following completion of the trial and database lock, in accordance with participant consent and institutional ethical requirements. Requests for clinical data should be directed to Professor Sarah Fidler (s.fidler@imperial.ac.uk) and will be reviewed according to the RIO trial data access policy. The RIO clinical protocol and Statistical Analysis Plan are available as supplementary documents accompanying the primary clinical publication (DOI: 10.1016/S2352-3018(26)00059-7). The generated research materials, including participant-derived HIV-1 env constructs, pseudovirus reagents, and other unique materials generated in this study, are available from the corresponding author upon reasonable request, subject to institutional approvals, material transfer agreements, participant consent restrictions, and material availability.

Research involving human participants, their data, or biological material

Policy information about studies with human participants or human data. See also policy information about sex, gender (identity/presentation), and sexual orientation and race, ethnicity and racism.

Reporting on sex and gender	All participants enrolled in the RIO trial were men. Sex was determined by self-report at enrollment. Gender identity was not collected separately. Because all enrolled participants were men, sex and gender were not considered as biological variables in the study design, and no sex- or gender-based analyses were performed.
Reporting on race, ethnicity, or other socially relevant groupings	Race and ethnicity were self-reported according to local clinical site procedures, they were collected for regulatory and safety reporting in the original RIO trial. These variables were not analyzed in the present study, did not inform inclusion/exclusion criteria, and were not used in any statistical analysis or interpretation. No conclusions were drawn based on race, ethnicity, or other socially constructed groupings.
Population characteristics	The population consisted of adults living with HIV-1 who initiated antiretroviral therapy (ART) during primary or early-stage infection and had maintained virologic suppression for ≥1 year prior to enrollment. Although the protocol permitted enrollment of both women and men, all enrolled participants were men. Baseline demographic characteristics are summarized in Supplementary Table 1. The majority had HIV clade B virus. Participants had well-preserved immunity (median baseline CD4 count 776 cells/μL) and had been on ART for a median of 5 years. These characteristics reflect the trial's focus on individuals with smaller and more homogeneous HIV reservoirs typical of early-treated infection.
Recruitment	Participants were recruited across nine sites in the UK and one in Denmark, as part of the RIO randomized controlled trial, using standard clinical research pathways within each participating National Health Service (NHS) or hospital system. Recruitment was restricted to individuals who met prespecified eligibility criteria (early or primary HIV infection, suppressed viral load, predicted sensitivity to bNAbs). Researchers involved in the present manuscript did not influence recruitment beyond the trial protocol, and only de-identified samples and data were used for the mechanistic analyses. Participants received financial compensation.
Ethics oversight	The RIO trial received ethics and regulatory approval in the United Kingdom from the London–Westminster Research Ethics Committee (REC reference 19/LO/1669; IRAS ID 266322), the UK Health Research Authority, and the Medicines and Healthcare products Regulatory Agency (MHRA reference 19174/0413/001). For the Danish study site, the trial received regulatory approval from the Danish Medicines Agency. The trial was registered under ClinicalTrials.gov (NCT04319367), the European Union Drug Regulating Authorities Clinical Trials (EudraCT) database (2019-002129-31), and the European Union Clinical Trials Regulation (EU CT number 2024-514564-13-00). All participants included in the present study were enrolled under these approved protocols and provided written informed consent before study participation. Trial conduct was overseen by the Imperial Clinical Trials Unit, with the Independent Data Monitoring Committee and Trial Steering Committee convening regularly to review participant safety and trial conduct. The analyses presented in this manuscript were performed using de-identified clinical samples and data collected under the approved study protocols.

Note that full information on the approval of the study protocol must also be provided in the manuscript.

Field-specific reporting

Please select the one below that is the best fit for your research. If you are not sure, read the appropriate sections before making your selection.

☒ Life sciences ☐ Behavioural & social sciences ☐ Ecological, evolutionary & environmental sciences

For a reference copy of the document with all sections, see nature.com/documents/nr-reporting-summary-flat.pdf

Life sciences study design

All studies must disclose on these points even when the disclosure is negative.

Sample size	Sample sizes for all analyses were determined by the availability of clinical samples collected within the RIO trial. No statistical methods were used to predetermine sample size for this mechanistic study. The exact number of participants or samples used in each experiment is reported in the corresponding figure legends and Methods sections.
Data exclusions	No data or samples were excluded from the analyses unless they failed prespecified and objective quality-control criteria (e.g., insufficient sequencing depth, non-amplifiable templates, or samples with <500 reads in the DIHIVA pipeline). These criteria were applied uniformly and are described in the Methods. No post-hoc exclusions were made.
Replication	All experimental assays included appropriate technical replicates as described in the Methods. Where biological replication was possible, samples from independent participants were analyzed. All reported findings were reproducible within the available biological samples, and no findings failed replication.
Randomization	Randomization was not applicable to the laboratory analyses in this study. Participants were randomized as part of the RIO clinical trial, but the present study performed mechanistic laboratory analyses on de-identified specimens and was not involved in participant allocation.
Blinding	All laboratory experiments were performed on de-identified samples labeled only with participant codes, with investigators unaware of clinical arm assignment at the time of analysis. Laboratory investigators performing the mechanistic analyses were initially blinded to treatment allocation. Blinding was maintained until participants met protocol-defined criteria for unblinding (for example, following viral rebound), after which treatment allocation became known for subsequent analyses

Reporting for specific materials, systems and methods

We require information from authors about some types of materials, experimental systems and methods used in many studies. Here, indicate whether each material, system or method listed is relevant to your study. If you are not sure if a list item applies to your research, read the appropriate section before selecting a response.

Materials & experimental systems

n/a	Involved in the study	
☐	☒	Antibodies
☐	☒	Eukaryotic cell lines
☒	☐	Palaeontology and archaeology
☒	☐	Animals and other organisms
☐	☒	Clinical data
☒	☐	Dual use research of concern
☒	☐	Plants

Methods

n/a	Involved in the study	
☒	☐	ChIP-seq
☒	☐	Flow cytometry
☒	☐	MRI-based neuroimaging

Antibodies

Antibodies used	This study used purified broadly neutralizing antibodies (bNAbs) 3BNC117 and 10-1074 for neutralization assays and autologous plasma neutralization experiments. No commercial antibodies were used for cell staining or flow cytometry in this work
Validation	The bNAbs 3BNC117 and 10-1074 have been extensively validated in multiple peer-reviewed clinical studies for binding specificity and neutralization breadth. No new antibody validation was performed for the present study.

Eukaryotic cell lines

Policy information about cell lines and Sex and Gender in Research

Cell line source(s)	TZM-bl cells were used for pseudovirus neutralization assays. Cells were obtained from the NIH AIDS Reagent Program.
Authentication	TZM-bl cells used for neutralization assays were obtained through the NIH HIV Reagent Program and are routinely monitored for expected assay performance through external quality assurance testing.

Mycoplasma contamination	TZM-bl Cell lines are routinely tested for mycoplasma contamination and were confirmed to be mycoplasma negative. No additional authentication (e.g., STR profiling or karyotyping) was performed.
Commonly misidentified lines (See ICLAC register)	TZM-bl cells are not listed among commonly misidentified or cross-contaminated cell lines in the ICLAC database. No other eukaryotic cell lines were used.

Clinical data

Policy information about clinical studies

All manuscripts should comply with the ICMJE guidelines for publication of clinical research and a completed CONSORT checklist must be included with all submissions.

Clinical trial registration	ClinicalTrials.gov: NCT04319367<br>European Union Drug Regulating Authorities Clinical Trials (EudraCT): 2019-002129-31<br>London–Westminster Research Ethics Committee (UK REC): 19/LO/1669<br>European Union Clinical Trials Regulation (EU CT): 2024-514564-13-00<br>Medicines and Healthcare products Regulatory Agency (MHRA): 19174/0413/001
Study protocol	The full clinical protocol and Statistical Analysis Plan are available as supplementary documents accompanying the primary clinical publication. (Lee MJ, et al. Time to HIV rebound after infusion of long-acting broadly neutralising antibodies 3BNC117-LS and 10-1074-LS and analytical treatment interruption (the RIO trial): a double-blind, randomised, placebo-controlled trial. Lancet HIV. 2026 Aug;13(8):e527-e537. doi: 10.1016/S2352-3018(26)00059-7. Epub 2026 May 27. PMID: 42202839.)
Data collection	Clinical data were collected prospectively between May 2021 and September 26, 2025, at participating HIV clinical research sites in the United Kingdom and Denmark following Good Clinical Practice (GCP) procedures. Data collection included viral load measurements, CD4 counts, pharmacokinetic sampling, and safety assessments. Samples for mechanistic assays (PBMCs, plasma, and serum) were collected at scheduled study visits, processed according to standardized procedures, and transferred as de-identified specimens for laboratory analyses.
Outcomes	The RIO trial prespecified time to viral rebound within 20 weeks after analytical treatment interruption as the primary clinical endpoint. Safety, delayed viral rebound, and prolonged ART-free viral control following analytical treatment interruption were pre-specified secondary in the trial protocol and reported previously. The present manuscript reports pre-specified secondary virologic outcomes, including reservoir quantification and baseline reservoir sensitivity to bNAbs, pre-specified exploratory analyses of viral sequencing, reservoir characterization, rebound virus evolution, and antibody correlates of viral control, as well as clearly identified post hoc analyses examining associations between reservoir characteristics and clinical outcomes. Clinical outcomes were prospectively assessed according to protocol-defined endpoint criteria using validated clinical and laboratory assays.

Plants

Seed stocks	*Report on the source of all seed stocks or other plant material used. If applicable, state the seed stock centre and catalogue number. If plant specimens were collected from the field, describe the collection location, date and sampling procedures.*
Novel plant genotypes	*Describe the methods by which all novel plant genotypes were produced. This includes those generated by transgenic approaches, gene editing, chemical/radiation-based mutagenesis and hybridization. For transgenic lines, describe the transformation method, the number of independent lines analyzed and the generation upon which experiments were performed. For gene-edited lines, describe the editor used, the endogenous sequence targeted for editing, the targeting guide RNA sequence (if applicable) and how the editor was applied.*
Authentication	*Describe any authentication procedures for each seed stock used or novel genotype generated. Describe any experiments used to assess the effect of a mutation and, where applicable, how potential secondary effects (e.g. second site T-DNA insertions, mosiacism, off-target gene editing) were examined.*
