# Supplementary material for: Broadly neutralizing antibodies in adult males living with HIV undergoing analytical treatment interruption: secondary and exploratory outcomes of the phase II randomized controlled RIO trial
Source: Nat Med. 2026 Sep 15;32(11):3990–4001. doi: 10.1038/s41591-026-04644-8 (PMC13768170; doi:10.1038/s41591-026-04644-8)
Supplement: Supplementary file 3 [file 41591_2026_4644_MOESM3_ESM.pdf]

nature portfolio

Peer Review File

Broadly neutralizing antibodies in adult males living with HIV undergoing analytical treatment interruption: secondary and exploratory outcomes of the phase II randomized controlled RIO trial

Corresponding Author: Professor Michel Nussenzweig

This manuscript has been previously reviewed at another journal. This document only contains information relating to versions considered at Nature Medicine.

This file contains all reviewer reports in order by version, followed by all author rebuttals in order by version.

Version 1:

Reviewer comments:

Reviewer #1

(Remarks to the Author)
the authors have address all the reviewers comments and have improved the manuscript by adding the reservoir data in group B and addressing all comments.
I have no further comments.

Reviewer #2

(Remarks to the Author)
I am satisfied that all my concerns have been addressed.

Reviewer #3

(Remarks to the Author)

In this revised analysis of the virology and humoral immunity in the RIO trial, the authors have done a good job responding to the prior concerns. Specifically, the authors have made the following changes below. Overall, the manuscript is significantly improved, although I still have some concerns as noted below.

- The authors developed a technique to deplete residual bNABs using anti-idiotypic antibodies (ext data Fig 3). The authors found no increase in aNAb activity against baseline viruses and no expansion of neutralization breadth.
- Performed a correlation analysis looking for baseline relationship between aNAb responses and HIV-specific T cell responses (Ext data Fig 8)
- No significant correlation between baseline reservoir size and aNAb activity (Ext data Fig 9)
- Performed a reservoir decay analysis of Arm A vs B (Fig 6, Ext Data Fig 11-12), including arm B reservoir size around week 52 before second ATI.
- The authors have fixed some of the inconsistencies noted in the manuscript

Some concerns that were raised were not fully addressed by the authors:
- The primary manuscript still has not been published and is only available as a preprint. I would hesitate to publish this secondary analysis before the primary paper has completed peer review. Problems identified during the review process of the primary study manuscript could affect the participants included in secondary analyses. If accepted, this manuscript could be published concurrently with the primary study results, provided there are no substantial changes to the primary manuscript that would affect this analysis.
- I noted that only about 1/3 of enrolled participants are included in the baseline reservoir env neutralization analysis. The

authors stated that “We now include all individuals for which we were able to recover reservoir sequences.” This statement vaguely implied that the authors had made additional attempts to perform sequencing. I was thus disappointed that a looking at the numbers in the manuscript, there was no change in the number of pseudoviruses tested or participants with aNAb data. I appreciate that it is challenging to obtain intact reservoir sequences from early-treated participants, but if they were not able to do so, then they should clearly state it in the Response to Reviewers instead of adding a vague statement that can be interpreted as being misleading and forcing the reviewer to spend time comparing numbers between manuscript versions.
- The authors should note that the vast majority of Rio participants (91% in arm A) were enrolled having met criteria for primary infection (as opposed to the early infection criteria)
- I appreciate that the authors developed a method to deplete bNAbs. It would be helpful to show that these bNAb-depleting protocol did not also deplete aNAbs. One simple way to do this is to take the 4 pseudotype viruses that were sensitive to baseline (pre-bNAb) IgGs as demonstrated by low IC80s in Extended data Figure 3a, and to deplete the baseline (pre-bNAb) IgG samples with the anti-idiotype antibodies. If the aNAb titers do not change with anti-idiotypic treatment of the IgG, this would be convincing evidence that this assay does not inadvertently deplete aNAbs. In addition, I had a difficult time finding some of the details of the bNAb depletion experiments, particularly the pre-depletion levels of residual bNAb levels and weeks for each participant.

Reviewer #4

(Remarks to the Author)
The authors have made a great effort in addressing the concerns raised during the initial review. They have performed new analyses, provided context, and clarified multiple aspects of the manuscript. However, limitations remain that hamper interpretation of the data and limit the significance of their findings. In particular, two key messages remain uncertain:

-Regarding the role of baseline autologous neutralizing activities: the authors argue that enhanced preexisting autologous neutralizing activities together with residual levels of infused bNAbs may contribute to maintaining viral control. This hypothesis makes sense. However, the sensitivity of reservoir virus to autologous antibodies correlates with sensitivity to 10-1074 (r=0.67, p=0.01 for arm A; r=0.57, p=0.005 for arm A+B), introducing a confounding variable that may explain the attenuation observed in their multivariable model. In other words, resistance to autologous neutralization may reflect an overall inherent difficulty in neutralizing this virus with 10-1074. Notably, participants with autologous IC80>500 had higher 10-1074 IC80 than others (p=0.015)

-Regarding the intact viral reservoir decay: to provide a contemporaneous reference, the authors now include arm B data, which showed no decay. However, this comparison is problematic, as arm B participants experienced viral replication between timepoints, potentially replenishing the reservoir. This is briefly mentioned by the authors in the discussion but remains nevertheless a significant concern. Less importantly, and likely a consequence of sample availability, 30% of arm B participants had undetectable baseline intact reservoir versus ~10% in arm A, which may somewhat confound comparisons

Reviewer #5

(Remarks to the Author)
The authors reported comprehensively protocol-specified secondary and exploratory analysis results from a placebo-controlled randomized ATI study where half of 68 participants received 2 bnAbs and half received placebo prior to ATI among participants who started ART during early stage HIV infection. The results are complex as the interplay between autologous antibodies, T-cells, bnAbs, antibody-mediated immune responses, reservoir viruses and rebounding viruses are dynamic and not completely understood. Overall, the authors did a decent job describing the study, the observed results and implications/interpretations. Here are some comments to improve clarity.
1) In the abstract: add the n’s for each arm. Clarify the direction (positive or negative) of the correlation between reservoir virus sensitivity to antibody and time to rebound.
2) “phylogenetic and antibody resistance analyses” are not a type of exploratory “outcome”. Reword.
3) Line #137: unclear whether all 15 Arm A and 11 Arm B participants contained at least 3 intact proviral sequences.
4) Line #140: unclear how the “representative” virus sequences were selected.
5) Line #144: were the <250 mcg/ml referring to that for the bnAbs, and < 1:100 dilution referring to autologous IgG measured using participant serum?
6) Line #151: missing “at”?
7) Reservoir viruses seemed to be more sensitive to the two bnAbs in Arm A than Arm B.
8) #166-168: since 7 and 2 participants’ time to resuming ART was censored at 96 weeks, it is appropriate to report the mean time without clarifying what those 9 observations contributed. Reporting the medians might be more appropriate. This comment also applies to the first paragraph of results on page 11, where it’s unclear how the censored values, if any, were handled in calculating the mean.
9) #182: unclear how the “representative” viruses were selected.
10) #243: was the cox analysis was done for Arm B participant examining the relationship between baseline reservoir autologous antibody sensitivity and time to ART restart?
11) #255: redundant word “therapy”?
12) Is figure 1d showing serum titer (i.e., inverse of the dilution factor of the serum sample) or IC80? If it’s the same as the other panels, why it’s not shown in a log-scale?
13) Figure 4: did not see 95% CI of the correlation coefficient as indicated in the figure legend.

14) #671: unclear whether time to ART restart was censored at any time or not. In the KM curves earlier, it seemed like it was censored at 96 weeks; however, in the later dot plots, it seemed like time beyond 96 weeks was observed. Also, good to clarify what analysis software was used for the cox analysis. What does “pairwise” analysis mean?

Version 2:

Reviewer comments:

Reviewer #3

(Remarks to the Author)
The authors have adequately addressed all of my previous comments and concerns. I believe that this revised manuscript satisfactorily resolves the major issues raised during review.
1. The primary study associated with this work has now been published.
2. The authors clarified that they obtained additional Env sequences [not confirmed intact proviral genome] and performed reservoir sensitivity testing against autologous antibodies.
3. They demonstrated that the bNAb depletion method did not significantly remove autologous antibodies.
4. They provided information regarding residual bNAb levels as well as the timing of post-infusion sample collection for each participant.

Since the authors performed experiments demonstrating that the bNAb depletion method did not significantly affect autologous antibodies, they could consider including it either as a supplemental figure, supplementary data, or a brief statement describing these findings to clearly address this concern for readers.

Reviewer #4

(Remarks to the Author)

In the revised version of the manuscript, the authors clearly acknowledge the limitations in the interpretation of the study's results. However, this does not resolve concerns regarding the manuscript's central conclusions: the link between bNAb infusion and reservoir decay , and the role of autologous antibodies (aNAbs)
Although it is likely that aNAbs and residual levels of bNAbs act together to maintain prolonged virus control, it is unclear whether residual bNAb levels alone could have achieved similar results. Although limited by small numbers, the authors' own results suggest that aNAb activity was not associated with better control in the placebo group. I think that the potential role of residual bNAb levels in maintaining viral control in participants carrying highly sensitive viruses is supported by the results at least as strongly as the possible role of aNAbs. This should be better reflected in the title and abstract of the manuscript.

Reviewer #5

(Remarks to the Author)
Impressive work. No additional comments.

Open Access This Peer Review File is licensed under a Creative Commons Attribution 4.0 International License, which permits use, sharing, adaptation, distribution and reproduction in any medium or format, as long as you give appropriate credit to the original author(s) and the source, provide a link to the Creative Commons license, and indicate if changes were made.
In cases where reviewers are anonymous, credit should be given to 'Anonymous Referee' and the source.
The images or other third party material in this Peer Review File are included in the article's Creative Commons license, unless indicated otherwise in a credit line to the material. If material is not included in the article's Creative Commons license and your intended use is not permitted by statutory regulation or exceeds the permitted use, you will need to obtain permission directly from the copyright holder.
To view a copy of this license, visit https://creativecommons.org/licenses/by/4.0/

Reviewers' Comments:

Reviewer #1 (Remarks to the Author):

the authors have address all the reviewers comments and have improved the manuscript by adding the reservoir data in group B and addressing all comments.
I have no further comments.

We thank the reviewer for the careful evaluation of our revised manuscript.

Reviewer #2 (Remarks to the Author):

I am satisfied that all my concerns have been addressed.

We thank the reviewer for the careful assessment of the revised manuscript.

Reviewer #3 (Remarks to the Author):

In this revised analysis of the virology and humoral immunity in the RIO trial, the authors have done a good job responding to the prior concerns. Specifically, the authors have made the following changes below. Overall, the manuscript is significantly improved, although I still have some concerns as noted below.

- The authors developed a technique to deplete residual bNABs using anti-idiotypic antibodies (ext data Fig 3). The authors found no increase in aNAb activity against baseline viruses and no expansion of neutralization breadth.
- Performed a correlation analysis looking for baseline relationship between aNAb responses and HIV-specific T cell responses (Ext data Fig 8)
- No significant correlation between baseline reservoir size and aNAb activity (Ext data Fig 9)
- Performed a reservoir decay analysis of Arm A vs B (Fig 6, Ext Data Fig 11-12), including arm B reservoir size around week 52 before second ATI.
- The authors have fixed some of the inconsistencies noted in the manuscript

We appreciate the reviewer's continued feedback.

Some concerns that were raised were not fully addressed by the authors:
- The primary manuscript still has not been published and is only available as a preprint. I would hesitate to publish this secondary analysis before the primary paper has completed peer review. Problems identified during the review process of the primary study manuscript could affect the participants included in secondary analyses. If accepted, this manuscript could be published concurrently with the primary study results, provided there are no substantial changes to the primary manuscript that would affect this analysis.

The primary manuscript is scheduled for publication on May 27th in Lancet HIV.

- I noted that only about 1/3 of enrolled participants are included in the baseline reservoir env neutralization analysis. The authors stated that "We now include all individuals for which we were able to recover reservoir sequences." This statement vaguely implied that the authors had made additional attempts to perform sequencing. I was thus disappointed that a looking at the numbers in the

manuscript, there was no change in the number of pseudoviruses tested or participants with aNAb data. I appreciate that it is challenging to obtain intact reservoir sequences from early-treated participants, but if they were not able to do so, then they should clearly state it in the Response to Reviewers instead of adding a vague statement that can be interpreted as being misleading and forcing the reviewer to spend time comparing numbers between manuscript versions.

We apologize for the lack of clarity. We have been unable to obtain additional intact proviral sequences despite a great deal of trying.

We have been able to obtain intact Env sequences from 5 additional participants from Arm A and 7 from Arm B, but we cannot be certain that these Envs represent intact viruses. Nevertheless, pseudovirus were produced and tested for sensitivity. We are reporting these additional sensitivities combined with our previous sensitivities from intact reservoir viruses as a new Extended data Fig. 6 and also incorporate in lines 235-239.

- The authors should note that the vast majority of Rio participants (91% in arm A) were enrolled having met criteria for primary infection (as opposed to the early infection criteria)

We thank the reviewer for pointing this out, the text has been revised accordingly.

- I appreciate that the authors developed a method to deplete bNAbs. It would be helpful to show that these bNAb-depleting protocol did not also deplete aNAbs. One simple way to do this is to take the 4 pseudotype viruses that were sensitive to baseline (pre-bNAb) IgGs as demonstrated by low IC80s in Extended data Figure 3a, and to deplete the baseline (pre-bNAb) IgG samples with the anti-idiotype antibodies. If the aNAb titers do not change with anti-idiotypic treatment of the IgG, this would be convincing evidence that this assay does not inadvertently deplete aNAbs.

We thank the reviewer for this excellent suggestion. This was also a concern for us, and to determine whether depletion with anti-idiotypic antibodies could also affect autologous neutralization, we performed a similar control experiment using purified IgG samples from two participants from the RIO trial (P32 and P48) and four participants from another ongoing trial (301, 138, 115, and 101) at later time points post-infusion of bNAbs (ranging from 38 to 116 weeks). The IgG samples were depleted or not for the bNAbs according to our protocol and neutralization tested against reservoir-derived pseudoviruses (results below). We observed no significant differences in autologous neutralization activity before and after depletion (maybe a tendency to decrease autologous neutralization instead, due to residual bNAb depletion), suggesting that the depletion protocol did not measurably remove autologous antibodies.

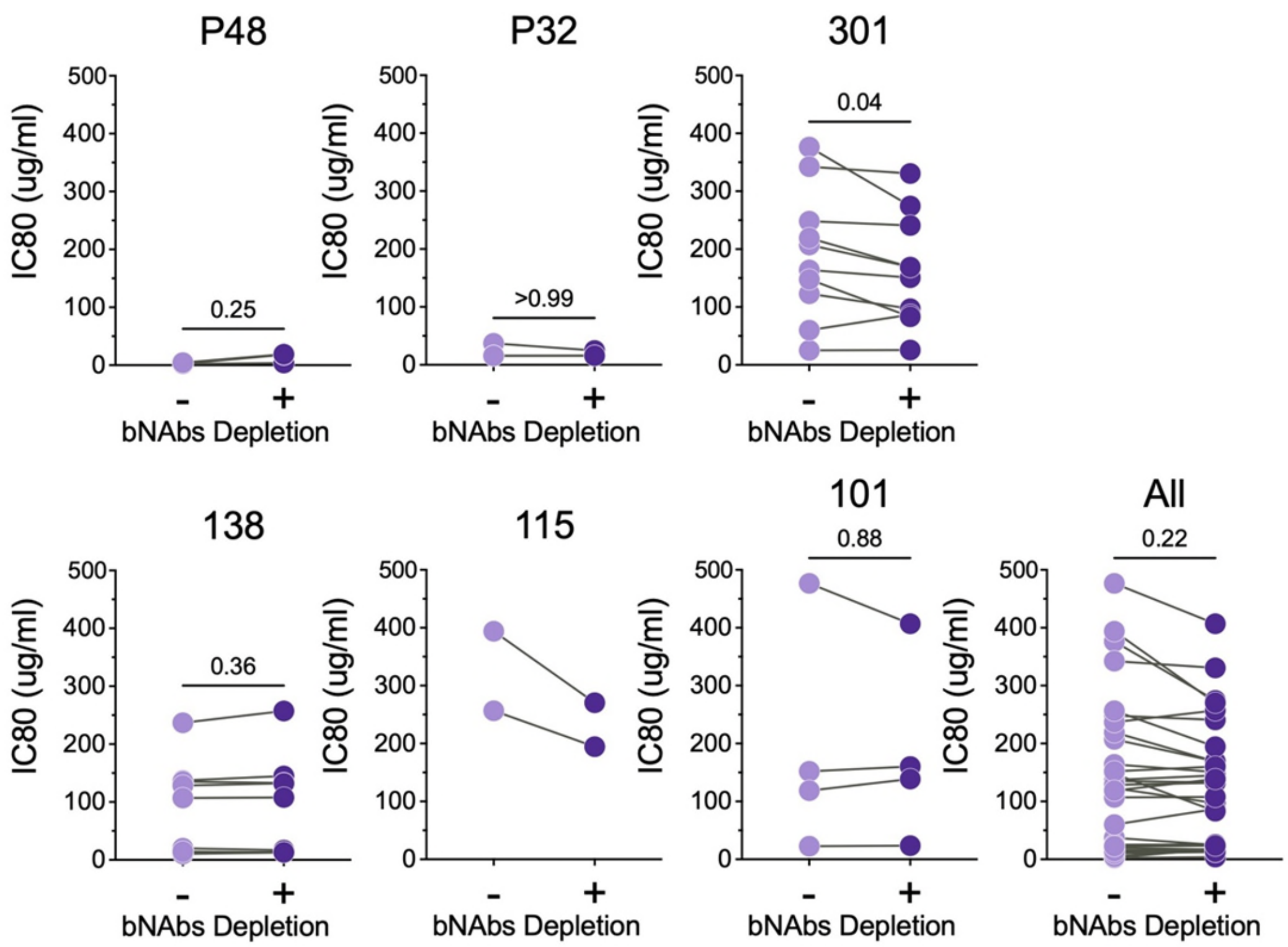

In addition, I had a difficult time finding some of the details of the bNAb depletion experiments, particularly the pre-depletion levels of residual bNAb levels and weeks for each participant.

We thank the reviewer to bring that to our attention. We have revised Extended Data Fig. 4 and its corresponding legend to include the week of post-infusion sample collection for each participant, as well as the residual plasma concentrations of 3BNC117 and 10-1074 measured before depletion of circulating bNAbs.

Reviewer #4 (Remarks to the Author):

The authors have made a great effort in addressing the concerns raised during the initial review. They have performed new analyses, provided context, and clarified multiple aspects of the manuscript. However, limitations remain that hamper interpretation of the data and limit the significance of their findings. In particular, two key messages remain uncertain:

-Regarding the role of baseline autologous neutralizing activities: the authors argue that enhanced preexisting autologous neutralizing activities together with residual levels of infused bNAbs may contribute to maintaining viral control. This hypothesis makes sense. However, the sensitivity of reservoir virus to autologous antibodies correlates with sensitivity to 10-1074 (r=0.67, p=0.01 for arm A; r=0.57, p=0.005 for arm A+B), introducing a confounding variable that may explain the attenuation observed in their multivariable model. In other words, resistance to autologous neutralization may reflect an overall inherent difficulty in neutralizing this virus with 10-1074. Notably, participants with autologous IC80>500 had higher 10-1074 IC80 than others (p=0.015)

This is a good point and we thank the reviewer for this thoughtful comment and agree that sensitivity of autologous IgGs and 10-1074 (or 3BNC117) positively correlate (See below panels a and c) and could be

a confounding variable that explains attenuation in the multivariable model. We emphasize this point in the revised text. Please, see line 253 – 256.

However, this correlation is mainly driven by a subset of participants showing no autologous neutralization (IC80 > 500). By removing this group, the significant correlation is abolished (panels b and d). In addition, finding that there is some correlation between autologous antibodies and bNAbs is somewhat expected since serologic reactivity is a composite of antibodies targeting different sites including sites targeted by bNAbs (10.1126/science.1207227 and 10.1038/nature10373).

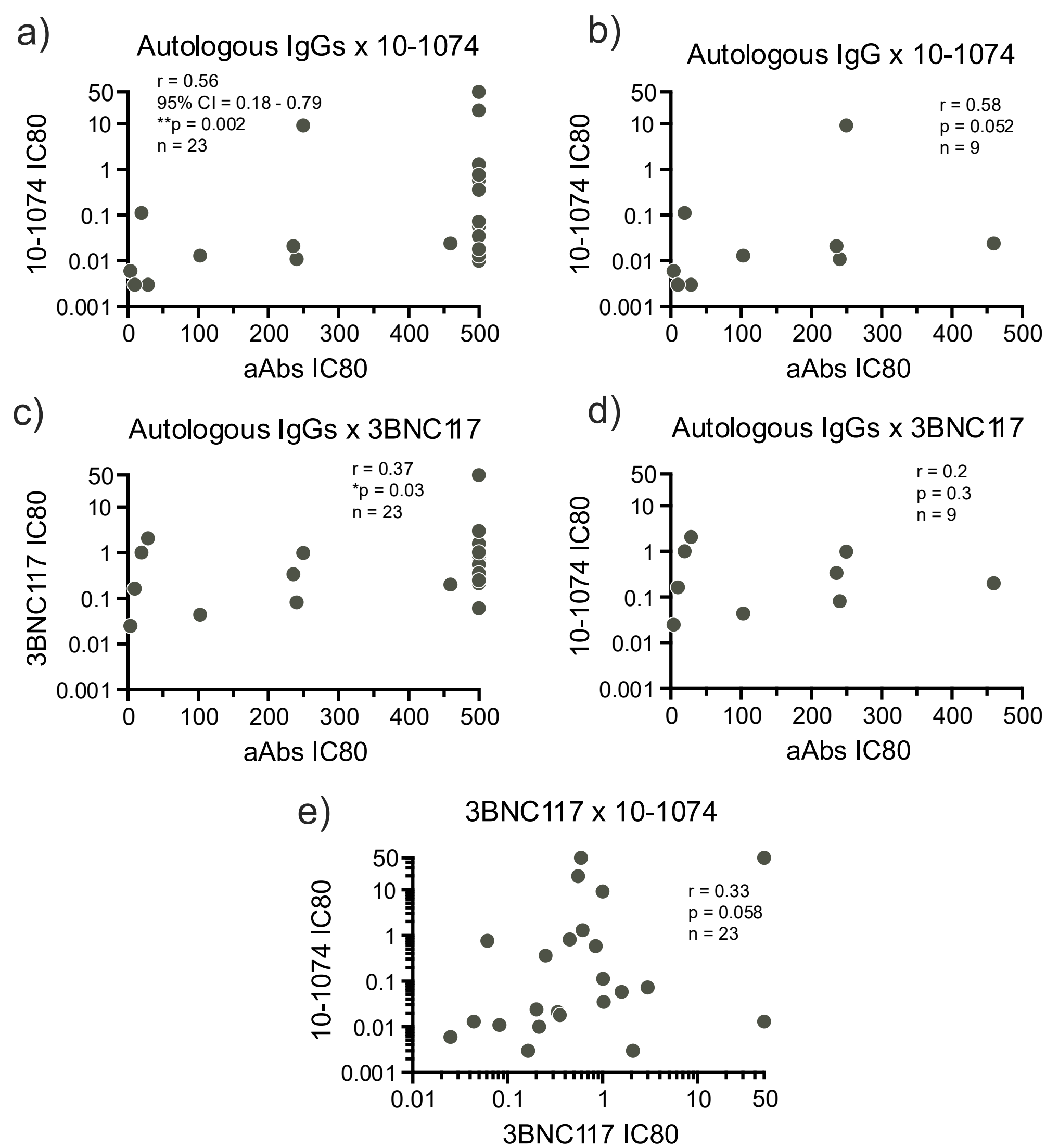

-Regarding the intact viral reservoir decay: to provide a contemporaneous reference, the authors now include arm B data, which showed no decay. However, this comparison is problematic, as arm B participants experienced viral replication between timepoints, potentially replenishing the reservoir. This is briefly mentioned by the authors in the discussion but remains nevertheless a significant concern. Less importantly, and likely a consequence of sample availability, 30% of arm B participants had undetectable baseline intact reservoir versus ~10% in arm A, which may somewhat confound comparisons

We thank the reviewer for raising this point and we agree that participants in Arm B experienced viral rebound and periods of detectable viremia between sampling time points, which could contribute to

increased detectable circulating reservoir and limit the direct comparison with Arm A. We have revised the Discussion to further emphasize this limitation and frame these findings more conservatively (Discussion lines 311-314).

Reviewer #5 (Remarks to the Author):

The authors reported comprehensively protocol-specified secondary and exploratory analysis results from a placebo-controlled randomized ATI study where half of 68 participants received 2 bnAbs and half received placebo prior to ATI among participants who started ART during early stage HIV infection. The results are complex as the interplay between autologous antibodies, T-cells, bnAbs, antibody-mediated immune responses, reservoir viruses and rebounding viruses are dynamic and not completely understood. Overall, the authors did a decent job describing the study, the observed results and implications/interpretations. Here are some comments to improve clarity.
1) In the abstract: add the n's for each arm. Clarify the direction (positive or negative) of the correlation between reservoir virus sensitivity to antibody and time to rebound.
2) "phylogenetic and antibody resistance analyses" are not a type of exploratory "outcome". Reword.
3) Line #137: unclear whether all 15 Arm A and 11 Arm B participants contained at least 3 intact proviral sequences.
4) Line #140: unclear how the "representative" virus sequences were selected.
5) Line #144: were the <250 mcg/ml referring to that for the bnAbs, and < 1:100 dilution referring to autologous IgG measured using participant serum?
6) Line #151: missing "at"?
7) Reservoir viruses seemed to be more sensitive to the two bnAbs in Arm A than Arm B.
8) #166-168: since 7 and 2 participants' time to resuming ART was censored at 96 weeks, it is appropriate to report the mean time without clarifying what those 9 observations contributed. Reporting the medians might be more appropriate. This comment also applies to the first paragraph of results on page 11, where it's unclear how the censored values, if any, were handled in calculating the mean.
9) #182: unclear how the "representative" viruses were selected.
10) #243: was the cox analysis was done for Arm B participant examining the relationship between baseline reservoir autologous antibody sensitivity and time to ART restart?
11) #255: redundant word "therapy"?
12) Is figure 1d showing serum titer (i.e., inverse of the dilution factor of the serum sample) or IC80? If it's the same as the other panels, why it's not shown in a log-scale?
13) Figure 4: did not see 95% CI of the correlation coefficient as indicated in the figure legend.
14) #671: unclear whether time to ART restart was censored at any time or not. In the KM curves earlier, it seemed like it was censored at 96 weeks; however, in the later dot plots, it seemed like time beyond 96 weeks was observed. Also, good to clarify what analysis software was used for the cox analysis. What does "pairwise" analysis mean?

We thank the reviewer for the careful evaluation of the revised manuscript and for the constructive suggestions to improve clarity and reporting of our study. We addressed each point as follow:

1. We revised the abstract including the number of individuals per Arm and clarifying the direction observed in our correlation between reservoir sensitivity and delayed rebound (Abstract lines 49–50 and 58).
2. We agree with the reviewer, these are analyses/methods not outcomes. We revised the wording (Results lines 120-121).

3. We clarified the number of participants containing at least three intact proviral sequences used for downstream analyses (Results lines 149-150).
4. We thank the reviewer for bringing this to our attention and we agree that the term “representative” require clarification. Pseudoviruses were not intentionally preselected based on neutralization phenotype or sequence characteristics. Instead, we attempted pseudovirus generation from all available distinct intact env sequences obtained from reservoir and included all constructs that produced viable infectious pseudoviruses suitable for TMZbl assay. We rephrased the results section to remove ambiguity (Results lines 159).
5. The IC80<250 ug ml-1 threshold refers to neutralization measured using purified autologous IgGs. This concentration corresponds approximately to 1:100 dilution of total serum IgG and was included only as a reference for physiological levels. We revised the wording accordingly for clarity (Results lines 162 - 164).
6. We corrected the typographical omission noted by the reviewer (Results line 170).
7. We agree that there is a tendency of lower sensitivity for both bNAbs in Arm A when compared to Arm B reservoir viruses. However, we didn’t observe significant differences when directly comparing both groups, and given the limited sample size and exploratory nature of this analysis, we decided to not overinterpret this observation in the current manuscript.
8. We thank the reviewer for raising this point and we agree that reporting mean times without explicitly addressing censoring may be misleading. As suggested by other reviewers, this paragraph was incorporated into the first paragraph from results section, into the ‘trial description’. We revised the text to clarify censoring at 96 weeks and now report medians where appropriate to avoid ambiguity (Results lines 112-114).
9. Same response as for question. n. 4. We revised the wording to avoid ambiguity. Thank you for bringing that to our attention.
10. The Cox proportional hazards analyses were performed only in Arm A participants, where prolonged delayed rebound under bNAb exposure was observed. We didn’t performed equivalent analysis in Arm B because no bNAbs were administered and no prolonged ART-free control was observed. We now mention that in line 250 as a clarification.
11. We removed the redundant wording noted by the reviewer (Results line 284).
12. We thank the reviewer for the comment. Figure 1d shows autologous IgG IC80 values rather than reciprocal serum dilution titers. We chose to present autologous neutralization data as linear IC80 values to facilitate visualization across the broader range of neutralization activity observed in this cohort. In contrast to the infused bNAbs, where small differences around low µg ml-1 concentrations are biologically informative and are better visualized using log axis, autologous neutralization values spanned a wider range, extending up to 500 µg ml-1.
13. We thank the reviewer for noticing that. We have included 95% coefficient interval on figures where correlations were performed.
14. We thank the reviewer for this important point. The reviewer is correct that Kaplan–Meier analyses in the accompanying clinical manuscript were censored at 96 weeks, and we did the same while mentioning in our manuscript. In contrast, for the exploratory correlation analyses presented in this manuscript, we used the observed time to ART restart for each participant, including events occurring beyond 96 weeks when available. We clarified this distinction in the revised Methods and additionally specified the statistical software used for Cox proportional hazards analyses and the meaning of pairwise comparisons (Methods lines 719 – 720 and 725-726).

Reviewer #3 (Remarks to the Author):
The authors have adequately addressed all of my previous comments and concerns. I believe that this revised manuscript satisfactorily resolves the major issues raised during review.
1. The primary study associated with this work has now been published.
2. The authors clarified that they obtained additional Env sequences [not confirmed intact proviral genome] and performed reservoir sensitivity testing against autologous antibodies.
3. They demonstrated that the bNAb depletion method did not significantly remove autologous antibodies.
4. They provided information regarding residual bNAb levels as well as the timing of post-infusion sample collection for each participant.
Since the authors performed experiments demonstrating that the bNAb depletion method did not significantly affect autologous antibodies, they could consider including it either as a supplemental figure, supplementary data, or a brief statement describing these findings to clearly address this concern for readers.

We thank the reviewer for all the constructive comments and suggestions. We have addressed this suggestion by including the depletion control experiment as Extended Data Fig. 4a and by adding a brief statement in the Results describing that the depletion procedure efficiently removed residual therapeutic bNAbs while preserving autologous antibody activity (lines 180–182).

Reviewer #4 (Remarks to the Author):
In the revised version of the manuscript, the authors clearly acknowledge the limitations in the interpretation of the study's results. However, this does not resolve concerns regarding the manuscript's central conclusions: the link between bNAb infusion and reservoir decay , and the role of autologous antibodies (aNAbs)
Although it is likely that aNAbs and residual levels of bNAbs act together to maintain prolonged virus control, it is unclear whether residual bNAb levels alone could have achieved similar results. Although limited by small numbers, the authors' own results suggest that aNAb activity was not associated with better control in the placebo group. I think that the potential role of residual bNAb levels in maintaining viral control in participants carrying highly sensitive viruses is supported by the results at least as strongly as the possible role of aNAbs. This should be better reflected in the title and abstract of the manuscript.

We have mentioned and strengthened the role of residual bNAbs in our manuscript. The title has been modified as Editorial suggestion. Please see the Abstract at lines 64 and the Discussion at lines 366-368 and 384.

Reviewer #5 (Remarks to the Author):
Impressive work. No additional comments.

We thank the reviewer for all the constructive comments and suggestions during the review process.

All best regards,

Michel
